# PSD95 nanoclusters are postsynaptic building blocks in hippocampus circuits

**DOI:** 10.1038/srep24626

**Published:** 2016-04-25

**Authors:** Matthew J. Broadhead, Mathew H. Horrocks, Fei Zhu, Leila Muresan, Ruth Benavides-Piccione, Javier DeFelipe, David Fricker, Maksym V. Kopanitsa, Rory R. Duncan, David Klenerman, Noboru H. Komiyama, Steven F. Lee, Seth G. N. Grant

**Affiliations:** 1 Centre for Clinical Brain Sciences (CCBS), University of Edinburgh, Edinburgh, United Kingdom; 2 Edinburgh Super-Resolution Imaging Consortium (ESRIC), Heriot Watt University, Edinburgh, United Kingdom; 3 Department of Chemistry, University of Cambridge, Lensfield Road, Cambridge, CB2 1EW. United Kingdom; 4 Cambridge Advanced Imaging Centre (CAIC), University of Cambridge, Cambridge, United Kingdom; 5 Instituto Cajal (CSIC) and Centro de Tecnología Biomédica (UPM), Madrid, Spain; 6 Synome Ltd, Babraham Research Campus, Cambridge, United Kingdom; 7 Institute of Biological Chemistry, Biophysics and Bioengineering, Heriot-Watt University, Edinburgh, EH14 4AS, UK

## Abstract

The molecular features of synapses in the hippocampus underpin current models of learning and cognition. Although synapse ultra-structural diversity has been described in the canonical hippocampal circuitry, our knowledge of sub-synaptic organisation of synaptic molecules remains largely unknown. To address this, mice were engineered to express Post Synaptic Density 95 protein (PSD95) fused to either eGFP or mEos2 and imaged with two orthogonal super-resolution methods: gated stimulated emission depletion (g-STED) microscopy and photoactivated localisation microscopy (PALM). Large-scale analysis of ~100,000 synapses in 7 hippocampal sub-regions revealed they comprised discrete PSD95 nanoclusters that were spatially organised into single and multi-nanocluster PSDs. Synapses in different sub-regions, cell-types and locations along the dendritic tree of CA1 pyramidal neurons, showed diversity characterised by the number of nanoclusters per synapse. Multi-nanocluster synapses were frequently found in the CA3 and dentate gyrus sub-regions, corresponding to large thorny excrescence synapses. Although the structure of individual nanoclusters remained relatively conserved across all sub-regions, PSD95 packing into nanoclusters also varied between sub-regions determined from nanocluster fluorescence intensity. These data identify PSD95 nanoclusters as a basic structural unit, or building block, of excitatory synapses and their number characterizes synapse size and structural diversity.

The structure and molecular composition of synapses in the hippocampus mediate its central role in learning and memory. The hippocampal formation receives information from neocortical sources and processes it in the sequential projections (classic trisynaptic hippocampal circuitry) connecting dentate gyrus (DG) granule cell neurons with the pyramidal cells of the CA3 and CA1 fields^1^. Electron microscopy (EM) has shown that there are differences in the size of the postsynaptic terminals between these sub-regions^2^,^3^,^4^,^5^ and that the size of terminals correlates with synaptic strength^6^,^7^,^8^,^9^. However, as EM studies of synaptic molecular organisation are limited by both indirect, non-covalent labelling (e.g. antibodies) and the relatively small number of synapses that can be studied (typically in the order of a few dozens or hundreds), little is known about the molecular substructure of different hippocampal synapses. More recently, fluorescence based super-resolution microscopy was demonstrated to be a powerful complementary approach for visualising molecular-based features of synapses in brain tissue^10^ and cultured neurons^11^,^12^,^13^,^14^. Since super-resolution methods can be applied to genetically labelled proteins, it offers the potential for the efficient studies of directly labelled endogenous synaptic proteins when combined with genome engineering in the mouse.

The accumulation of postsynaptic proteins is a prominent feature of the sub-synaptic anatomy and can be readily observed with EM and super-resolution microscopy. EM studies first defined the electron-dense accumulation in excitatory synapses as the postsynaptic density (PSD)^15^,^16^,^17^. The composition of the PSD has been characterised with proteomic methods and found to contain over 1000 proteins^18^, and one of the most abundant proteins is Post Synaptic Density 95 (PSD95)^19^,^20^. EM of PSD95 labelled with immuno-gold particles showed it was distributed throughout the PSD^21^,^22^,^23^. Super-resolution studies of transfected cultured neurons show PSD95 was concentrated into nanoclusters (NCs) of approximately 50–250 nm in diameter^11^,^12^,^13^. Some synapses contained multiple NCs suggesting that NCs may be subdomains of the PSD and their number was a determinant of the PSD size.

Presently little is known about PSD95 nanostructure in mature neurons in brain tissue and this is likely to be important for reasons beyond the understanding of the hippocampal circuitry. PSD95 is a scaffold protein and central organiser of postsynaptic signalling complexes comprising glutamate receptors, ion channels, signalling enzymes and adhesion proteins^24^,^25^. Since these complexes are ~2 MDa in size^26^, the PSD95 NCs must represent local packing of dozens or hundreds of these complexes. The organisation of multiple NCs into individual synapses would represent a further level of higher-order organisation at the sub-synaptic scale. It is therefore important to document the diversity of NCs in different synapses of the mature hippocampus and ask if multi-NC synapses exist *in vivo* and if they are found in particular cell-types or synapses in the hippocampal circuitry. Moreover, PSD95 is of major functional importance as it controls synaptic transmission, synaptic plasticity and learning and memory^19^,^27^,^28^,^29^ and PSD95 complexes are mutated in schizophrenia, autism and intellectual disability^24^,^25^,^29^,^30^,^31^,^67^,^68^. Thus, elucidation of the neuroanatomical diversity of PSD95 NCs in the hippocampus will further our understanding of mechanisms of cognition and mental illness.

Here we report a study of the nano-architecture of synapses in the hippocampal formation by combining the technology of genetically modified mice, where endogenous PSD95 was labelled with fluorescent proteins, with two orthogonal forms of super-resolution microscopy. We used mice that were genetically engineered to express PSD95 fused to either eGFP or mEos2 (PSD95-eGFP, PSD95-mEos2), which are suitable for use with two orthogonal super-resolution methods: gated stimulated emission depletion (g-STED) microscopy and photoactivated localisation microscopy (PALM). Using these cross-validating approaches and highly quantitative methods, we performed the first systematic large-scale study of PSD95 nanoarchitecture across the hippocampal formation in brain sections from mature mice. Through a study of over 100,000 PSDs, we confirm the presence of NCs *in vivo* and find they are a universal and highly conserved unit or building block underpinning the structure of simple and complex PSDs. We found characteristic anatomical distributions of PSD95 NCs in distinct hippocampal regions, cell-types and locations within the dendritic tree. Large, complex synapses such as the thorny excresences of CA3 pyramidal neurons were found to contain multiple NCs. This study demonstrates the power of combining investigations of synapse nano-architecture with large-scale anatomical approaches to define basic mechanisms of synapse structure. The tools and approaches described here have wide application *in vitro* and *in vivo*.

## Results

### Genetically modified mice labelling endogenous PSD95

We previously reported the generation of a line of knock-in mice that allowed us to perform highly efficient purification of synaptic proteins, their complexes and studies of synaptic protein localisation across the brain^25^. In that mouse line, tandem affinity purification (TAP) tags were fused in-frame to the carboxyl terminus of endogenous PSD95 using gene targeting and we have used the same strategy with eGFP and mEos2 tags, hence expressing the fusion proteins under the control of PSD95’s regulatory elements. Here we describe the PSD95-mEos2 mice (Supplementary Fig. 1a), the PSD95-eGFP mice are described elsewhere (Zhu F *et al*. in preparation). Low magnification (×20) fluorescence slide scanning reveals that PSD95-eGFP and PSD95-mEos2 mice have the same expression pattern of PSD95 across the brain with high levels of expression in the cortex, striatum and the hippocampus (Supplementary Fig. 1b). Confocal microscopy (×100) in the CA1 stratum radiatum (CA1^SR^) of the hippocampus, showed that PSD95-eGFP and PSD95-mEos2 form puncta of approximately 300–600 nm diameter (Supplementary Fig. 1c). Staining both PSD95-eGFP and PSD95-mEos2 brain sections for a presynaptic marker (synaptophysin I) revealed presynaptic puncta juxtaposed with PSD95 as expected (Supplementary Fig. 1c). In the PSD95-eGFP mice the expression level of the tagged protein was comparable to that of wild type (untagged) protein (Zhu F *et al*. in preparation). However, we noticed that the expression level of the tagged protein was reduced in PSD95-mEos2 mice (Supplementary Fig. 1d). Electrophysiological studies of homozygous and heterozygous PSD95-mEos2 mice showed least phenotypes in heterozygous mice and they were therefore used for imaging studies (Supplementary Fig. 1e,f). PSD95-eGFP mice showed no changes in electrophysiological properties (data not shown) and homozygous mice were used for g-STED imaging.

### Super-resolution imaging of endogenous tagged PSD95 in brain

**Figure 1:**
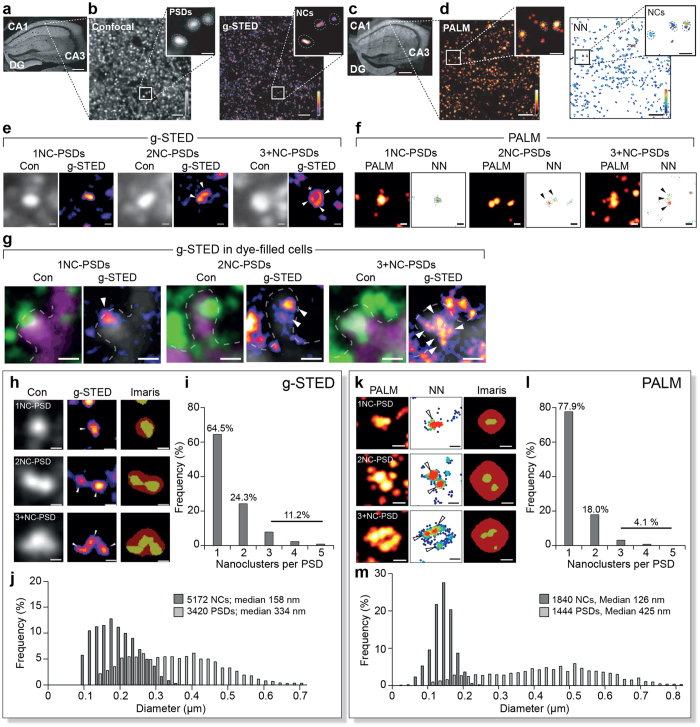
Super-resolution reveals PSD95 nanostructure and synaptic diversity in brain tissue. (**a**) Coronal section of the hippocampus from a PSD95-eGFP mouse. CA1, CA3 and dentate gyrus (DG) regions are shown. Scale bar 250 μm. (**b**) Left panel, confocal image from the CA1^SO^ shows PSD95eGFP puncta, corresponding to PSDs (expanded inset). Scale bar 2 μm, inset images scale bars 500 nm. Right panel, correlative g-STED image reveals NCs. (**c**) Coronal section of the hippocampus from a PSD95-mEos2 mouse. CA1, CA3 and dentate gyrus (DG) regions are shown. Scale bar, 250 μm. (**d**) Left panel, PALM image from the CA1^SO^ of PSD95-mEos2 sections. Scale bars 2 μm, inset images scale bars 500 nm. Right panel, image rendered with nearest neighbour (NN) analysis reveals structures showing significant sub-clustering indicating NCs. (**e**) Examples of synapse subtypes defined by the number of NCs (white arrowheads) per PSD observed using g-STED. Left panel corresponds to the confocal image and right panel corresponds to the super-resolved image. Asterisk indicates multiple NCs in a ring-like conformation. Scale bars 200 nm. (**f**) Synapse subtypes defined by the number of NCs (black arrows) per PSD, observed using PALM. Scale bars 200 nm. (**g**) Dye-filled neurons show single (1NC-PSD) and multiple NCs (2NC-PSD, 3+NC-PSD) in spine heads. Left panel, PSD95-eGFP (green) imaged with a confocal (Con) microscope and the Alexa Fluor 594-filled spine is shown in magenta. Right panel, g-STED image of PSD95-eGFP in same spine. Scale bars 500 nm. White arrowheads, NCs; magenta, Alexa Fluor 594 dye; dotted line, estimated spine membrane location. (**h**) Example of segmentation of g-STED images of PSDs (red area) and NCs (yellow area) using Imaris Cell. (**i**) Frequency (%) histogram of the number of NCs per PSD from 3420 PSDs from the CA1^SO^. (**j**) Frequency (%) histogram of the diameter of 5172 NCs and 3420 PSDs from the CA1^SO^. (**k**) Example of segmentation of PALM images of PSDs (red area) and NCs (yellow area) using Imaris Cell. (**l**) Frequency (%) histogram of the number of NCs per PSD from 1444 PSDs analysed from the CA1^SO^. (**m**) Frequency (%) histogram of the diameters of 1840 NCs and 1444 PSDs from the CA1^SO^.

We first asked if PSD95 was organised into NCs in the brain of our two mouse models. As shown in Fig. 1, PSD95-eGFP sections were imaged using sequential confocal (lateral resolution 240 nm) and g-STED microscopy (lateral resolution 85 nm) (see Supplementary Fig. 2 for g-STED resolution measurements). Confocal images showed a characteristic punctate (PSD) distribution of PSD95, which was further resolved into nanoclusters using g-STED (Fig. 1a,b). Similarly, PSD95 NCs were observed using PALM imaging (localisation precision ~40 nm, see Supplementary Fig. 3) from brain sections of PSD95-mEos2 (Fig. 1c,d). Although the PSD was originally an EM definition, the punctate synaptic imaging of PSD95 with light microscopy is frequently used as a definition of the PSD, so hereafter we refer to the PSD as the PSD95 distribution within the confines of a single puncta. Both forms of super-resolution microscopy revealed that most PSDs contained a single NC whereas some contained multiple NCs (Fig. 1e,f). Dye filling of neurons confirmed single and multiple NCs were within single spine heads (Fig. 1g). An initial manual quantification (FWHM, full width half maximum analysis of line profiles) in the CA1 stratum oriens (CA1^SO^) of ~200 PSD95-eGFP NCs showed a median diameter of 136 nm and an elliptical shape (86 × 189 nm median short and long axes) (Supplementary Fig. 4a–d). These results are consistent with previous reports from primary cultures of rat hippocampus neurons^11^,^12^,^13^.

To exploit our mouse models with their high number of labelled synapses and potential anatomical diversity, we developed automated methods within Imaris Cell that detect, segment and quantify PSDs and their respective NCs (Fig. 1h–m). The analysis settings were assessed for robustness using a combination of Monte Carlo simulated data (Supplementary Fig. 4e–g) and manually detected PSD95-eGFP structures (Supplementary Fig. 4h,i). Similarly, manual quantification validation was performed for PSD95-mEos2 NCs (Supplementary Fig. 4j–o).

Using these methods we first analysed the CA1^SO^ and found that the median diameter of their PSDs was 334 nm (g-STED, n = 3420) and 425 nm (PALM, n = 1444) and that of NCs was 158 nm (g-STED, n = 5172) and 126 nm (PALM, n = 1840) (Fig. 1j,m). From these data, it was determined that 1NC-PSDs were most abundant (g-STED: 63.7 ± 1.6%, PALM: 75 ± 2.6%) while those PSDs with greater numbers of NCs were increasingly rare; 2NC-PSDs (g-STED: 24.3 ± 0.4%, PALM: 19 ± 1.4%), and 3+NC-PSDs (g-STED: 11.9 ± 1.2%, PALM: 5.6 ± 1.4%) (Fig. 1i,l). These results, obtained by using two mouse models and two forms of super-resolution microscopy provide the first quantification of NCs in intact mouse brain tissue.

### Diversity of PSD95 nanostructure in the hippocampus

**Figure 2:**
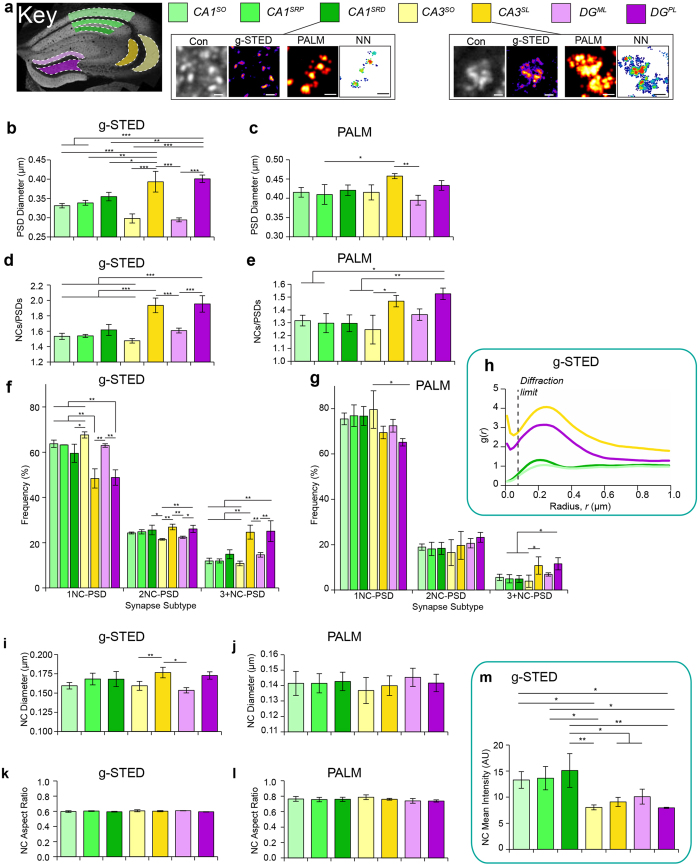
PSD95 nanostructural diversity between hippocampal sub-regions. (**a**) A colour-coded key to the seven hippocampal sub-regions surveyed, a coding which is maintained for the subsequent panels b–m. CA1 sub-regions are shown in shades of green, CA3 sub-regions in yellow and DG sub-regions in magenta. Image panels from CA1^SRD^ show typical 1NC-PSDs and CA3^SL^ multi-NC-PSDs. Con, confocal; NN, nearest neighbour. Scale bars 500 nm. (**b**) PSD95-eGFP PSD diameter. (**c**) PSD95-mEos2 PSD diameter. (**d**) PSD95-eGFP NC/PSD. (**e**) PSD95-mEos2 NC/PSD. (**f**) PSD95-eGFP frequency histogram of synapse subtypes. (**g**) PSD95-mEos2 frequency histogram of synapse subtypes. (**h**) Paired Correlation Function (g(r)) analysis from PSD95-eGFP NCs. Denotes the probability of clustering of NCs at a given radius, r. Dotted line denotes the diffraction limit of g-STED. (**i**) PSD95-eGFP NC diameter. (**j**) PSD95-mEos2 NC diameter. (**k**) PSD95-eGFP NC aspect ratio. (**l**) PSD95-mEos2 NC aspect ratio. (**m**) Mean fluorescence intensity of PSD95-eGFP NCs. *P < 0.05; **P < 0.01; ***P < 0.001.

The scalability of our methods enabled us to examine key brain regions and ask if there is diversity in the nanoarchitecture of synapses in important circuits. To this end, we performed a comprehensive quantitative super-resolution survey of the trisynaptic circuit of the hippocampus focusing on three main regions: dentate gyrus (DG), CA3 and CA1. These regions were further subdivided into a total of seven sub-regions: DG, molecular layer (DG^ML^), polymorphic layer (DG^PL^); CA3, stratum oriens (CA3^SO^), stratum lucidum (CA3^SL^); CA1, stratum radiatum proximal (CA1^SRP^), stratum radiatum distal (CA1^SRD^) and stratum oriens (CA1^SO^) (Fig. 2a). A total of 89,452 NCs within 55,617 PSDs from three PSD95-eGFP mice were studied using g-STED and 25,017 NCs within 18,763 PSDs from three PSD95-mEos2 mice using PALM (Supplementary Tables 1 and 2). Multiple further examples of images from these regions are presented in Supplementary Figs 5 and 6.

We first examined the PSD size and found significant differences between sub-regions with g-STED (*F* = 31.4, *P* = 2.4 × 10^−7^) and PALM (*F* = 4.7, *P* = 0.0082). We observed a striking dichotomy within the two sub-regions of both DG and CA3, with one sub-region showing the largest and the other showing the smallest PSDs (Fig. 2b,c). The CA3^SL^ and DG^PL^ displayed the largest PSDs (g-STED diameter of 398 ± 22 nm and 405 ± 10 nm respectively, mean ± SD) and these likely correspond to characteristically large PSDs of the thorny excrescence synapses found in these regions^3^ (see insets in Fig. 2a, Supplementary Figs 5e,g and 6e,g). Multi-NC-PSD of the thorny excrescences were also observed from dye-filled neurons (Supplementary Fig. 7a–d). In contrast, the CA3^SO^ and DG^ML^ shared the smallest PSDs (319 ± 7 nm and 322 ± 4 nm respectively, mean ± SD). Unlike the sub-regions of the CA3 and DG, the CA1 sub-regions showed no statistically significant difference in the size of their PSDs, although there was a trend for PSDs to increase in size from the stratum oriens to the distal stratum radiatum (CA1^SO^, 341 ± 6 nm; CA1^SRP^, 345 ± 2 nm; CA1^SRD^, 360 ± 9 nm).

To understand these differences in the PSD size we next examined the number of NCs, their size and shape within the sub-regions (Fig. 2d–m). A significant difference in the number of NCs per PSD (NC/PSD) was observed between sub-regions in both g-STED (*F* = 27.7, *P* = 5.4 × 10^−7^) and PALM (F = 7.4, *P* = 1.4 × 10^−6^). Moreover, there was a direct correspondence between the PSD size and NCs/PSD: the largest PSDs (CA3^SL^ and DG^PL^) showed the highest NCs/PSD (1.93 ± 0.10 NC/PSD and 1.95 ± 0.11 NC/PSD respectively) and the regions containing smaller PSDs (CA1^SO^ and CA3^SO^) showed the lowest number of NCs/PSD (1.53 ± 0.04 and 1.48 ± 0.03 respectively) (Fig. 2d,e). To examine the differential distribution of NCs between sub-regions in greater detail, we counted the number of PSDs containing one, two or more NCs in each sub-region (Fig. 2f,g). This clearly illustrates the significant dichotomy in NC numbers between sub-regions of CA3 and DG. To validate these findings using an orthogonal method and provide information on the distance between NCs, we next performed paired correlation function analyses on the g-STED data. By calculating the distance between any given structure and its nearest neighbouring structure, their spatial organisation can be represented by the value *g*(*r*) over a given radius (*r*). In all sub-regions, the maximal *g(r)* range was 100–300 nm (Fig. 2h; Supplementary Fig. 8) and as expected, the CA3^SL^ and DG^PL^ PSDs containing multiple NCs showed the greatest degree of NC clustering (both in terms of peak g(*r*) value and the radius over which clustering was significant (Fig. 2h; Supplementary Fig. 8e,g). We further validated these findings using manual quantification of PSD95 NCs in dye-filled neurons in CA1, CA3, DG and neocortex (Supplementary Fig. 7, Supplementary Table 3). Together these results show that specific sub-regions within the hippocampus contain PSDs with discrete numbers of PSD95 NCs.

We next asked if the differences in PSD size were also a function of the size of individual NCs (Fig. 2i,j). PALM data showed no significant differences (*F* = 0.4, *P* = 0.84) in NC diameter between sub-regions, with all approximately 140 nm. Similarly, g-STED data revealed that only 2 out of 21 sub-regional pairings displayed significant differences in NC diameter; namely NCs of the DG^ML^ (154 ± 4 nm) were smaller than NCs in the CA3^SL^ (177 ± 7 nm; *P* < 0.008) and DG^PL^ (173 ± 5 nm; *P* < 0.032). In addition, it was shown that NCs did not change in their elliptical morphology between sub-regions, as calculated by their mean average aspect ratio in both g-STED (mean aspect ratio 0.60; *F* = 1.5, *P* = 0.23, Fig. 2k) and PALM (mean aspect ratio 0.75; *F* = 1.2, *P* = 0.36, Fig. 2l). These findings indicate that NC size and shape were very similar between sub-regions and independent of PSD size.

Although the size and shape of NCs appeared to be conserved, we found evidence suggesting regional molecular diversity of NCs with respect to the amount of PSD95 per NC. The mean fluorescence intensity of PSDs and NCs (from g-STED data) showed significant inter-regional differences (PSDs: *F* = 21.3, *P* = 2.4 × 10^−5^, Supplementary Fig. 9a; NCs: *F* = 8.6, *P* = 0.0005, Fig. 2m, Supplementary Fig. 9b). Specifically, PSDs and NCs of the three CA1 sub-regions were all found to be of greater mean fluorescence intensity than those expressed in other hippocampal sub-regions. Given that the size and shape of NCs were conserved, this could indicate denser packing of PSD95 molecules in CA1 NCs. Due to a significant variation between the PSD95-mEos2 brain sections, however, the mean density of localisations in PSDs and NCs, as detected by PALM, was not different between sub-regions (PSDs: F = 0.6, P = 0.762, Supplementary Fig. 9c; NCs: F = 0.4, P = 0.845, Supplementary Fig. 9d,e). Together these findings indicate that NCs are ‘units’ of PSD organisation and their number underpins synapse diversity with distinct regional molecular variety. Furthermore, the data indicates that non-random allocation of NCs characterises the specific populations of PSDs distributed in brain regions.

### PSD95 nanostructure in CA1 pyramidal cells

**Figure 3:**
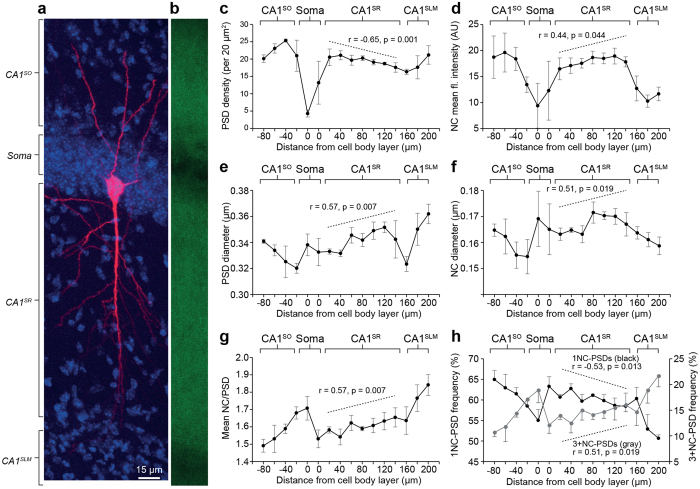
Diversity in PSD95-eGFP nanostructure within the CA1 radial gradient. (**a**) A CA1 injected pyramidal neuron with dendritic arborisations extending basally into the CA1^SO^, and apically through the CA1^SR^ and terminating in the CA1^SLM^. (**b**) Fluorescence expression of PSD95eGFP in the radial gradient of the hippocampus captured at low magnification (20x) from the same microscopic field as shown in (**a**). (**c**) PSD density (per 20 μm^2^) plotted as a function of distance from the CA1 soma layer. Different sub-fields of the CA1 are denoted with grey bars above the graph. Dotted line describes the Pearsons correlation analysis within the CA1^SR^. (**d**) Mean fluorescence intensity of NCs as a function of distance from the CA1 soma layer. (**e**) PSD diameter as a function of distance from the CA1 soma layer. (**f**) NC diameter as a function of distance from the soma layer. (**g**) Mean number of NCs per PSD as a function of distance from the CA1 soma layer. (**h**) Fractional population (%) of 1NC-PSDs (black line, left axis) and 3+NC-PSDs (grey line, right axis) plotted as a function of distance from the soma layer.

To explore the potential for this model as a mechanism of synapse diversity within single neurons, we next focused on CA1 pyramidal cells. CA1 pyramidal neurons project basal dendrites into the CA1^SO^ and apical dendrites into the CA1^SR^ extending into the CA1 stratum lacunosum moleculare (CA1^SLM^) (Fig. 3a). Each of these dendritic sub-regions receive synaptic inputs from different circuits^32^,^33^,^34^. It is unknown whether there are differences in PSD95 nanostructure across the CA1 pyramidal neuron dendritic tree. To address this, we analysed sets of g-STED images at regular intervals spanning the apical and basal dendritic tree (Fig. 3a,b). In total, 28,263 NCs were quantified within 17,467 PSDs from three mouse brain sections.

There was a complex patterning in PSD95 distribution and sub-synaptic nanostructural parameters in the CA1 (Fig. 3c–h). Not only were there sharp changes in puncta density and intensity between the CA1^SO^, CA1^SR^ and CA1^SLM^, but there were also gradual changes as a function of distance from the soma, particularly across the CA1^SR^ (Fig. 3c–h). Pearson’s correlation analysis was conducted over the range of 20–140 μm (seven 20 μm steps) from the soma layer into the CA1^SR^ to test for changes in the density of PSDs (Fig. 3c) and NCs, mean fluorescence intensity (Fig. 3d), size of PSDs (Fig. 3e) and NCs (Fig. 3f) and the number of NCs per PSD (Fig. 3g,h). The PSD density showed a strong negative correlation with distance in CA1^SR^ (*r* = −0.65, *P* = 0.001) with 19.2 ± 1.7 PSDs (per 20 μm^2^) at 20 μm distance from the soma and 16.5 ± 1.4 PSDs (per 20 μm^2^) at 140 μm (Fig. 3c). While the number of PSDs was reduced further from the soma, there was an increase in the mean fluorescence intensity of NCs (*r* = 0.44, *P* = 0.044; Fig. 3d). Moreover, it was found that the average size of PSDs increased along the same gradient, from 333 ± 2 nm diameter to 342 ± 14 nm over the same distance range (*r* = 0.57, *P* = 0.007; Fig. 3e). These findings demonstrate a shift in expression of PSD95 within the CA1^SR^ whereby proximal dendrites express numerous small synapses and distal dendrites express fewer, larger synapses expressing more PSD95.

As expected from our earlier findings, a positive correlation in the average number of NCs per PSD was observed as a function of distance from the soma (*r* = 0.57, *P* = 0.007; Fig. 3g). At 20 μm from the soma, the average number of NC/PSD was 1.58 ± 0.02, while at 140 μm distance, the PSDs contained an average of 1.65 ± 0.06 NCs (Fig. 3g). This finding is also reflected in Fig. 3h, which illustrates fractional populations of 1NC-PSDs and 3+NC-PSDs. There is a negative correlation in the percentage of 1NC-PSDs with distance (60.7 ± 0.6% to 58.5 ± 3.2%; r = −0.53, *P* = 0.013) and a positive correlation of 3+NC-PSDs with distance (13.9 ± 0.7% to 16.0 ± 1.3%; *r* = 0.51, *P* = 0.019) (Fig. 3h). The population for 2NC-PSDs did not change over this distance range (25.4 ± 1.0% to 25.5% 3.0%; *r* = 0.17, *P* = 0.462). Surprisingly, NCs had a greater diameter as a function of distance from the soma over the CA1^SR^ (*r* = 0.51, *P* = 0.019; Fig. 3f). This correlation seemed to be attributable to an increase in NC diameter from 163 ± 3 nm to 172 ± 4 nm at 80 μm from the soma. At 120 μm from the soma, there was a gradual reduction in NC diameter to 159 ± 3 nm in the CA1^SL^ (Fig. 3f). In addition to these findings in the CA^SR^, there were distance-dependent changes in NC parameters in both CA1^SO^ and CA1^SLM^. These findings indicate that NC diversity occurs across the dendritic tree of CA1 pyramidal neurons.

### PSD95 NCs are conserved between synapse subtypes

If PSD95 NCs were a basic structural unit of excitatory synapses, then we might expect they would show conserved features across a diverse range of synapse subtypes and anatomical regions. Our large datasets afford an unprecedented opportunity to address this issue. Using both the g-STED and PALM data sets, PSDs and NCs were sub-categorised by the synapse subtype to which they belonged (1NC-PSD, 2NC-PSD or 3+NC-PSD) and diameter and integrated fluorescence intensity (or number of localisations with PALM measurements) of quantified NCs and PSDs.

**Figure 4:**
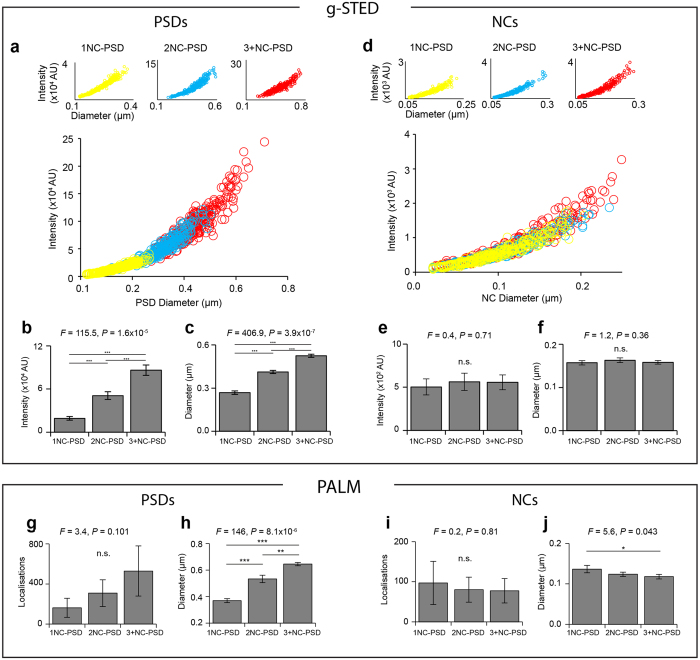
Quantitative analysis of PSD95 building block principle. Panels a–f show quantifications from g-STED data. (**a**) Scatter plots of the diameter and integrated fluorescence intensity of 600 randomly selected PSDs from the CA1^SO^ with 200 PSDs from each of the three synapse subtypes plotted in their own respective colour coded plots (upper graph) and plotted together (lower graph). Not only is there a correlation in size and total fluorescence intensity (as expected), but with higher number of NCs per PSD, the size and intensity of PSD fluorescence also become larger. Thus, PSDs in synapses of different subtypes can be characterised by their size and intensity. (**b**) Histogram of PSD integrated intensity values in different synapse subtypes. (**c**) Histogram of PSD diameters in different synapse subtypes. (**d**) Scatter plots of the diameter and integrated fluorescence intensity of 600 randomly selected NCs from the CA1^SO^ with 200 NCs from each of the three synapse subtypes plotted in their respective colour coded plots (upper graph) and plotted together (lower graph). While there is an expected positive correlation between the size and integrated fluorescence intensity of NCs, little difference is observed between the three populations. Thus, NCs belonging to different synapse subtypes are similar structures. (**e**) Bar chart of the integrated intensity of NCs from different synapse subtypes. (**f**) Bar chart of the diameter of NCs from different synapse subtypes. Panels g–j show quantifications from PALM data. (**g**) Histogram of total PSD localisations in different synapse subtypes. (**h**) Histogram of PSD diameters in different synapse subtypes. (**i**) Histogram of the total NC localisations in different synapse subtypes. (**j**) Histogram of NC diameters in different synapse subtypes.

From g-STED data, it was found that the size (*F* = 407, *P* = 4 × 10^−7^) and integrated fluorescence intensity of PSDs (*F* = 116, *P* = 2 × 10^−5^) positively correlated with the number of NCs per PSD (Fig. 4a–c). In the CA1^SO^, PSDs with 1 NC averaged 269 ± 12 nm in diameter, 2NC-PSDs had a diameter of 412 ± 10 nm and 3+NC-PSDs had a diameter of 525 ± 11 nm (Fig. 4c). When the diameters and intensities of NCs were compared between synapse subtypes, however, it was found that NCs did not vary significantly in their size (*F* = 1.2, *P* = 0.36) or integrated fluorescence intensity (*F* = 0.37, *P* = 0.71) (Fig. 4d–f). This analysis was further performed for PSDs and NCs of each of the other six hippocampal sub-regions (Supplementary Fig. 10). Every sub-region showed the same basic organisational principle – the PSD size in different subtypes of synapses depended on the number of NCs contained, while the individual NCs within each synapse subtype remained conserved in their size and fluorescence intensity (Supplementary Fig. 10).

The same analysis was then performed across the PALM data set, quantifying the diameters and the total number of localisations of PSD95 in PSDs and NCs from the same subtypes of synapses (Fig. 4g–j). Consistent with the above g-STED findings, the size of PSD95-mEos2 PSDs positively correlated with the number of NCs, and there was also a trend for increased numbers of total localisations (Fig. 4g,h). We found that the diameter of PSD95-mEos2 NCs was slightly reduced in those synapse subtypes with higher numbers of NCs per PSD (Fig. 4j). In the CA1^SRP^ for example, NCs in 1NC-PSDs had an average diameter of 137 ± 8 nm, while NCs within 2NC-PSDs and 3+NC-PSDs were lower: 123 ± 4 nm (*P* < 0.017) and 118 ± 3 nm (*P* < 0.041) respectively (Fig. 4j). This analysis was also performed on six other hippocampal sub-regions (Supplementary Fig. 11). Overall, considering the extent of statistical significance, it is concluded that NCs likely remain conserved in their overall structure and that structural synaptic diversity is defined by the number of NC ‘building blocks’ per PSD.

## Discussion

The combination of mouse genetic engineering and super-resolution microscopy has enabled us to carry out the first large-scale systematic study of the organisation of an endogenous protein in the mammalian brain at the nano-structural scale. With these methods, fundamentally important observations were made in quantifying the nanoscale features, organisation and distribution of a single protein population. Using two mouse models and super-resolution methods, we have established and cross-validated a set of robust methods for studying the molecular organisation of the PSD and its constituent NCs. A key advantage of our approach was the scalability, which afforded systematic analysis of synaptic nano-architecture across the synapses in the circuits of the hippocampal formation. We examined ~100,000 PSDs, which is several orders of magnitude greater than previous reports using EM^3^,^4^,^35^, thus providing robust datasets for statistical analyses of PSD diversity. These methods could be readily deployed in any other brain region, with the potential to make a nanoscale map of all brain regions, as well as being utilised in studies of development, behaviour, pharmacology and disease.

A striking and patterned diversity was observed using molecular labelling of PSD95, a major postsynaptic protein widely used as a marker of PSDs. This diversity was evident between regions, sub-regions, cell types and locations along the dendritic tree. In contrast to the diversity in PSDs, we found that the constituent NCs were of similar dimensions and that the number of NCs per PSD was the principal determinant of PSD diversity. Categorising synapses according to their numbers of NCs (1NC, 2NC, 3+NC PSDs) provided a basis for asking whether the different subtypes of PSDs were randomly distributed or found in specialised cells or regions. PSDs in all categories were found in all regions and in both CA3 and DG we found a dichotomy of PSD subtypes; one sub-region within each of these areas contained high numbers of large multi-NC PSDs and the adjacent sub-region contained low numbers with smaller PSDs. Since the DG is comprised of granule cells and CA3 of pyramidal neurons, we conclude that the differential distribution of PSDs with different numbers of NCs is not determined by the neuronal subtype. Because pyramidal neuron synapses in the CA3^SL^ and CA3^SO^ had distinct populations of multi-NC synapses and these receive distinct presynaptic inputs^33^,^36^, it suggests that PSD diversity and the mechanism responsible for distributing the number of NCs, is linked to presynaptic identity. Similarly, the stark contrast in fluorescence intensity of PSD95-eGFP NCs between the CA1^SR^ and the CA1^SLM^ may relate to the differential inputs received by CA1 pyramidal cells in these two subregions of the same dendritic tree^32^. Since the nanocluster size is invariant, it follows that the molecular specification of synapse formation does not influence the number of PSD95 molecules in a nanocluster. This suggests that cell-autonomous mechanisms determine nanocluster size and cell non-autonomous mechanisms determine nanocluster number and PSD95 copy number. Moreover, it is likely that these two mechanisms are sequentially activated^37^,^38^,^39^: the fibre pathways in the hippocampus are established before PSD95 protein expression arises in late postnatal development (~P14) and thus the cell non-autonomous molecular signals instruct the specification of PSD95 NC number and the cell-autonomous mechanisms produce NCs of invariant size. Several cell-autonomous mechanisms have been identified that control PSD95 localisation including palmitoylation^13^,^40^ and a potential cell non-autonomous mechanism is ligand activation of Ephrin3B^41^. Future studies will be required to address the role of these mechanisms in establishing the nanocluster formation and diversity in the hippocampus.

The functional significance of the nanoscale molecular architecture can be considered at the level of individual synapses and circuits. PSD95 is known to play a key role in controlling both synaptic strength^42^,^43^ and synaptic plasticity of AMPA receptors at excitatory synapses^44^,^45^. Consistent with our observations of the distribution of PSD95 NCs in the dendritic tree of pyramidal neurons in CA1^SR^, electrophysiological studies show that synaptic strength is dependent on the distance from the soma^46^,^47^ and thought to be mediated by the increased AMPA receptor number per synapse. Our observations in the CA1^SR^ in PSD95 NCs suggest a strong correlation between AMPA receptor number and NC number per PSD, suggesting a mechanism for PSD95 NCs in regulating synaptic strength. Evidence from super-resolution of PSD95 in cultured neurons also suggests that synaptic scaling increases synapse strength through increased number of NCs per PSD^11^. Previous EM studies have linked the existence of larger ‘complex’ PSDs with increased synaptic activity^6^,^48^, protein turnover rates^49^ and increased learning^35^,^50^. Greater numbers of PSD95 NCs indicates greater numbers of PSD95 protein complexes, which are known to be diverse in composition and control many signalling functions^24^,^25^. Hence larger PSDs and multi-NC synapses may also have more sophisticated plasticity functions.

From our data we can discern that there must be at least two different factors influencing PSD95 organisation into NCs. Firstly, a significant increase in the number of PSD95 molecules at the synapse increases the number of NCs rather than the size of the NC itself. This is in agreement with data of Nair *et al*.^12^ who showed that overexpression of PSD95 increased the number of NCs per PSD, but not their average size. Secondly, our data suggests that all NCs are not all equivalent and there is a molecular diversity within the NCs. We observed that the number of PSD95 molecules per NC varied in NCs from different regions. A potential explanation for this arises from insights into the organisation of PSD95 multiprotein complexes, which must be packed into NCs. Proteomic studies show >100 proteins interact with PSD95^24^,^25^,^26^,^51^ and these are known to have differential regional distribution^52^. There are approximately ~300 copies of PSD95 per synapse^11^,^12^,^19^ and we estimate that each NC contains 50–100 PSD95 protein complexes. Collectively, these facts suggest that PSD95 levels regulate numbers of NCs, and PSD95 interactions with binding partners (within signalling complexes) determines the composition, and hence, functional properties of NCs. The packing of signalling complexes into NCs would confer properties such as signal amplification, specificity and spatial control.

The model that PSD95 NCs are units or building blocks of PSDs provides a simple model for the construction of both simple and complex PSDs described previously by EM. We found that the large, complex thorny excrescence synapses correspond to multi-NC PSDs. Multi-NC PSDs sometimes appear to form ring-like formations (Fig. 1e) that may represent perforated or fenestrated PSDs described in EM studies^17^,^49^,^53^. Similarly, 2NC-PSDs may represent partitioned PSDs − an intermediate between single and complex perforated PSDs, whereby each NC aligns with a presynaptic release site within the same presynaptic bouton^6^. Alternatively, some multi-NC-PSDs could represent multi-innervated PSDs which receive synaptic inputs from different presynaptic boutons^35^,^54^. Future studies using correlative EM and super-resolution may clarify the molecular structure of these nano-structural features. Although we have demonstrated that sampling of large numbers of PSD95 structures in 2D can generate a representative view of its structure through averaging, 3D g-STED or PALM imaging would allow a more thorough visual examination of nanostructures. An interesting feature of the multi-NC PSDs was that the mean distance separating NCs was 100–300 nm. We speculate that there must be structural mechanisms, such as cytoskeletal proteins or lipid bilayer components, determining this spacing as well as regulating the conserved size of NCs. Understanding the mechanisms that partition PSD95 molecules into the structural hierarchy from individual protein molecules, to multiprotein complexes, NCs and whole PSDs will be facilitated by our mouse models and the super-resolution approaches. These tools will also be directly applicable to developmental and genetic studies and probing the mechanisms of the many diseases involving PSD95 complexes and the PSD.

## Methods

### Mouse Generation and Genotyping

All mouse procedures were performed in accordance with UK Home Office regulations and approved by Edinburgh University Director of Biological Services. Both PSD95-eGFP and PSD95-mEos2 fluorescent knockin mice were constructed using the same method and targeting strategy as described by Fernandez *et al*.^25^. The generation of the PSD95-eGFP knock-in mice are specifically described by Zhu *et al*., (in preparation) and the generation of PSD95-mEos2 mice is described here. We have shown the PSD95eGFP faithfully recapitulates the endogenous PSD95 protein levels, assembly into supercomplexes, anatomical localisation, developmental timing of expression and electrophysiological function in the hippocampus CA1-CA3 synapses. We used the previously constructed PSD95 intermediate targeting vector created in the generation of PSD95 TAP-tag mice^25^. The mEos2-C1 coding sequence was a gift from Michael Davidson^55^ (Supplementary Fig.1a).

Briefly, eGFP and mEos2 coding sequences were inserted into the open reading frame of PSD95/*Dlg4* gene at the 3′ end immediately before its stop codon using recombineering in *Escherichia coli* as described in previously published methods^56^ (Supplementary Fig.1a). Targeting plasmid DNAs were extracted using XhoI and BglII endonuclease restriction sites and electroporated into mouse embryonic stem cells. Positive targeted ES cell clones were identified by long-range polymerase chain reaction (PCR), cloned, expanded and frozen down before their injection into blastocysts from C57BL/6J mice. Backcrosses between adult chimaeric males and wildtype females generated heterozygous mice that were intercrossed to produce homozygous mice. Tail DNA was extracted and analysed by PCR with an f’ PSD95F5 primer and two 3′ pneoR4 and PSD95R6 primers to distinguish the PSD95 wild-type alleles (+/+) from either the heterozygous (+/eGFP; +/mEos2) or homozygous alleles (eGFP/eGFP; mEos2/mEos2).

### Electrophysiological Recordings and Data Analysis of PSD95-mEos2 mice

Electrophysiological recordings in the CA1 region of hippocampal slices in homozygous PSD95-mEos2 mice were used to test for any functional consequences of the mEos2 tag insertion. Acute hippocampal slices were prepared from 5–11 month old animals following previously published standard procedures^57^. Field excitatory postsynaptic potentials (fEPSPs) were recorded by the MEA60 electrophysiological suite (Multi Channel Systems, Reutlingen, FRG). To record fEPSPs, a hippocampal slice was placed into the well of a 5×13 3D MEA biochip (Qwane Biosciences, Lausanne, Switzerland). Monopolar stimulation of Schäffer collateral/commissural fibres through array electrodes was performed by a STG4008 stimulus generator. Biphasic (positive/negative, 100 μs/a phase) voltage pulses were used. Amplitude, duration and frequency of stimulation were controlled by the MC_Stimulus II software. We performed all long-term potentiation (LTP) experiments using two-pathway stimulation of Schäffer collateral/commissural fibres^58^. A single principal recording electrode in the middle of proximal part of CA1 was chosen and two electrodes were assigned for stimulation of the control and test pathways on the subicular side and on the CA3 side of stratum radiatum respectively. The distance from the recording electrode to the test stimulation electrode was 420–510 μm and to the control stimulation electrode 280–447 μm.

To evoke orthodromic fEPSPs, stimulation electrodes were activated at a frequency of 0.02 Hz. Peak amplitude of the negative part of fEPSPs was used as a measure of the synaptic response. Following at least 10–15 min of equilibration period inside an MEA well, I/O relationships were obtained and baseline stimulation strength was set to evoke a response that corresponded to ~40% of the maximal attainable fEPSP at the principal recording electrode. Paired pulse facilitation (PPF) was observed after stimulating Schäffer collateral/commissural fibres with a pair of pulses at baseline stimulation strength and an interpulse interval of 50 ms. PPF value was calculated as fEPSP_2_/fEPSP_1_*100%. Average data from five paired-pulse stimulations were used for each slice. LTP was induced after 60 min period of stable baseline responses by the theta-burst stimulation (TBS) train consisting of 10 bursts given at 5 Hz with 4 pulses given at 100 Hz per burst. Stimulus strength was not altered during LTP induction. LTP plots were scaled to the average of the first five baseline points. To account for a possible drift of baseline conditions, peak values in the test pathway were normalised by peak amplitudes in the control pathway prior to statistical comparison. LTP magnitude was assessed by averaging normalised fEPSPs in the test pathway 60–65 min after LTP induction.

Since several slices were routinely recorded from every mouse, values of area under the I/O relationship (AUC_I/O_), PPF and LTP in wild-type and mutant mice were initially compared using two-way nested ANOVA design with genotype (group) and mice (sub-group) as fixed and random factors correspondingly (STATISTICA v. 10, StatSoft, Inc., Tulsa, OK, USA). To contrast data obtained in PSD95^+/mEOS2^ and PSD95^mEOS2/mEOS2^ mutants with measurements performed in partly litter-matched WTs, we used *post hoc* Dunnett’s Multiple Comparison test on data from individual slices or on averaged data from several slices for each mouse if nested ANOVA reported a significant sub-group effect. Statistical effects were considered significant if *P* < 0.05. Graph plots and normalisation were performed using OriginPro 8.5 (OriginLab, Northampton, MA, USA). For electrophysiology data only, data are presented as the mean ± standard error of the mean with *n* and *N* indicating number of slices and mice respectively.

### Histology

Two month year old adult mice were anaesthetised with an intraperitoneal injection of 20 mg Euthatal (Merial Animal Health). Mice were perfused with 1× phosphate buffered saline (PBS; Oxoid), followed by 4% v/v paraformaldehyde (PFA; Alfa Aesar). Whole brains were dissected out and post-fixed in 4% PFA for 3–4 hours at 4 °C then washed out and replaced with 33% w/v sucrose solution (VWR Chemicals) in 1× PBS for 2–3 days. Brains were then embedded into a cryomould of the OCT compound (VWR) and frozen with liquid nitrogen. Cryosections were prepared at 18 μm thickness, mounted on slides and stored at −80 °C. The sections imaged were between bregma −1.6 and −2.2 mm. For PSD95-eGFP samples, coverslips were mounted over sections using home-made MOWIOL with DABCO. For PALM, sections were washed with 0.02 μm filtered 1× PBS and adhered to an argon plasma cleaned, poly-L-lysine (Sigma-Aldrich) coated coverslips with a small volume of filtered 1× PBS. Sections were placed for 1 h in darkness with the coverslip face down to aid adherence to the coverslip.

### Cell Filling, Immunohistochemistry

Cell filling was performed as described in previous methods^59^,^60^. Briefly 200 μm sections were cut from a 4% PFA perfused fixed brain using a vibratome and cells were injected with a continuous current of Alexa Fluor 594 dye solution (Life Technologies) in CA1, CA3 and DG region of the hippocampus. This fluorescent marker was applied, with the aid of an electrode, by continuous current until the distal tips of each dendrite fluoresced brightly, thereby ensuring that the dendrites were completely filled and the fluorescence did not diminish at a distance from the soma. For immunohistochemistry, brain sections were blocked and permeabilised in 5% bovine serum albumin (BSA, Sigma-Alrich) and 0.2% Triton X-100. Anti-synaptophysin I (1:250, ab14692, Abcam) used in solution of 3% BSA, 0.2% Triton X-100 at 4 °C overnight. Secondary antibody incubation of Alexa Fluor 594 (1:500, A-11012, Life Technologies) was performed for 1 hour at room temperature. Sections were then washed in 1x PBS and mounted.

### Low Magnification Scanning and Confocal Microscopy

Low magnification brain section scans were acquired with a Zeiss Axio Scan Slide Scanner (Carl Zeiss) with a ×20 plan-apochromat lens at 488 nm excitation with the same 500 ms exposure time. Hemi-coronal section images were background and contrast adjusted to provide reasonable comparison of expression. Both were compared with a WT negative control brain. Maps of PSD95-eGFP and PSD95-mEos2 were captured using x40 magnification confocal images with the Leica SP5 with individual images stitched together using Adobe Photoshop. High magnification x63 confocal images of PSD95-eGFP and Synaptophysin were captured using a Zeiss LSM510 with 46 × 46 × 130 nm pixel dimensions in accordance with Nyquist sampling capturing six serial z-plane acquisitions. Images were subsequently deconvolved with Huygens deconvolution software prior analysis. High magnification confocal images of PSD95-mEos2 and Synaptophysin were captured using the Leica SP5 at 20 × 20 × 130 nm pixel dimensions with six serial z-plane acquisitions. Low magnification images of dye filled cells in PSD95-eGFP sections were captured using a Zeiss LSM 710 with a x20 plan-apochromat lens. High magnification images of PSD95 antibody stains were captured using a Ziess LSM 510 with a x63 plan apochromat lens.

### g-STED Microscopy

Images were acquired using a Leica SP5 SMD g-STED microscope available at the Edinburgh Super-Resolution Imaging Consortium (ESRIC) hosted by Heriot-Watt University. Excitation was provided from a CW super-continuum white light laser source at 488 nm for eGFP samples, and the fluorescence signal was acquired using the Leica Hybrid detector between 500–560 nm and gated between 2–8 ns. We used a ×100 1.4NA STED objective lens and optical zoom set to provide 20 × 20 nm pixel dimensions. 2D confocal and g-STED images were acquired serially, with the confocal image taken first followed by the g-STED image.

Stimulated emission depletion was achieved using a 592 nm depletion laser. The optimal depletion laser power was calibrated using 22.5 ± 0.5 nm diameter P22 virus capsids expressing internal GFP, kindly donated by Prof. Trevor Douglas^61^. Capsids were adhered to a slide precoated in Cell-Tak^TM^ (Corning). P22 capsids were analysed by fitting Gaussian curves to line profiles drawn along two perpendicular axes to obtain the average full width at half maximum (FWHM) for 8–10 capsids at each depletion laser power. A depletion laser power density of 58 MW/cm^2^ measured prior to the objective lens can optimally resolve capsids at ~90 nm.

### PALM

PALM images were recorded on a bespoke home-built instrument consisting of an Olympus IX71 inverted microscope with an infinity-corrected oil immersion objective using TIRF illumination (Olympus UPlanApo TIRF, ×60, 1.49 NA) and detected on a 512 × 512 pixel EMCCD camera (Photometrics Evolve, EVO-512-M-FW- 16-AC-110). The brain sections were adhered close to a coverslip using poly-L-lysine to aid TIRF illumination of the sample. Images were acquired at 33 ms per frame operating in frame transfer mode with an election multiplication gain of 250 fold. We used a dichroic mirror (Semrock, Di01-R488/561) and a long pass filter (Semrock, LP02-568RS-25) designed for imaging the photo-converted form of the mEos2 fluorophore. Images were acquired through excitation at 561 nm (∼4 kW/cm^2^) for 18000 frames and photoactivated with 405 nm laser bursts (~10 W/cm^2^) every 300 frames. Laser intensity modulation was achieved using mechanical shutters (Prior, Optiscan III, V31XYZEF). The microscope hardware and image acquisitions were steered using open source microscopy manager software (Micro Manager 1.4). We determined the xy resolution of PALM as a function of the localisation fitting precision and the average nearest neighbour value per detected NC in accordance with previously published methods^11^,^62^.

### Image Pre-Processing

For g-STED data, images were processed using a Gaussian kernel prefilter (0.04 um) to enable effective peak detection, which is also used in displays of Confocal/g-STED images. Single-molecule events were localised using the GDSC Single Molecule Light Microscopy plugin Peak Fit in ImageJ (Alex Herbert, University of Sussex, UK) by fitting to a 2D Gaussian function using the Least Squared Errors method, displayed with their average fitting precision (40 nm). Using a previously published approach^11^ clusters of PSD95-mEos2 single molecule localisation events, indicative of whole PSDs, were defined using DBSCAN^63^ compiled in the statistics package R (version 3.1.2). Nearest neighbourhood (NN) analysis was then performed on localisations within their respective cluster determined from DBSCAN and pixel values were weighted on the NN coefficient, and localised spots displayed as 40nm circles to represent average fitting precision.

### Image Analysis

FWHM analysis of nanoclusters was performed in ImageJ using an open access code to fit line profiles to a Gaussian curve and calculate the FWHM from the fit (John Lim, March 2011). Line profiles were drawn along structures manually within ImageJ using a straight line drawing tool. Typically two lines were drawn perpendicular to one another, crossing at the estimated centre of the structure of interest. Where an elliptical structure was noticeable, the two line profiles would be drawn across the longest and shortest determinable axes. Diameters were calculated as the mean average of the two FWHM measurements.

Super-resolution image analysis was performed using Imaris Cell (Imaris v7.6.5 and v8.0.1). Specific parameters within the software were determined using a combination of FWHM analysis of raw images for g-STED (Supplementary Fig. 4a–d) and PALM (Supplementary Fig. 4j,k) data sets, Monte Carlo simulations to test robustness (Supplementary Fig. 4e–g) and assessment of % true detections *vs* manual quantifications (Supplementary Fig. 4h,i,l–o).

PSD95-eGFP PSDs were detected from confocal images and NCs from g-STED images using background subtraction with a diameter of 0.14 μm. Watershed based object splitting is then performed with both PSDs and NCs using a set seed point diameter of 0.4 μm and 0.16 μm respectively as determined from assessment against manual quantifications (Supplementary Fig. 4h–i). PSDs and NCs containing less than 30 and 10 pixels respectively, and structures segmenting XY border, were excluded. ~10% PSDs did not contain a detectable nanocluster, and were filtered from the final analysis.

Detection of PSD95-mEos2 PSDs and NCs was performed in a similar manner to g-STED. The whole cluster of the PSD was detected from the NN-rendered images with a large 0.25 μm Gaussian kernel blur and the same background subtraction value. For NC detection, NN-images were first smoothed with a Gaussian filter of 0.04 μm in accordance with the average precision fitting (Supplementary Fig. 3). NC detection was then performed with a background subtraction value of 0.07 μm based on manual FWHM analysis of unprocessed PALM images fitted and localized to their pixel centroids (Supplementary Fig. 4j,k). Watershed splitting for PSDs and NCs was performed with seed point diameters of 0.25 μm and 0.07 μm respectively, the latter of which tested vs manual quantification as shown in Supplementary Fig. 4l–o.

Juxtaposition analysis between PSD95 fluorescence and synaptophysin I was performed using Imaris Spots to detect synaptic puncta followed by object based colocalization analysis. Puncta within 600 nm of each other (centre to centre) were deemed juxtaposed. As a control to asses whether there was a significant association between the pre and postsynaptic puncta, analysis was also run on the same data in which one image channel was rotated 90° clockwise.

Analysis of PSD95-eGFP NCs and PSDs within dye filled spines was quantified manually to detect structures within spine head realms. Spine measurements were quantified manually in ImageJ by drawing lines across the widest aspect of the spine head and also from the base of the spine to tip of the head to determine length.

### Spatial Cluster Analysis of Nanoclusters

Paired-correlation function analysis was performed using custom compiled code in R using Spatstat^64^. The X-Y homogenous centre of mass coordinates of NCs were taken from one representative image from each of the sub-regions from each mouse. The paired correlation function, *g(r)*, was plotted over a radial distance of 1 μm *(r)*. The envelopes are simulated by generating 49 poisson processes for the same window of observation and taking the extrema for each *(r)*^65^. Values of *g(r)* greater than the confidence envelopes for a given *r* value were deemed significantly more clustered than random.

### Biochemical studies

Mouse forebrains were homogenised in a 2 mM HEPES 7.4 pH buffer containing 320 mM sucrose, phosphatase inhibitors (Merck Millipore) and EDTA-free protease inhibitors (Roche). Synaptosomes were purified similar as described by a standard protocol^66^. Denatured samples were loaded into Precast Nupage Bis-tris gradient gels (Life Technologies) for electrophoresis at 200 V continuous current for 60 min and then transferred to a nitrocellulose membrane for immunoblotting. Membranes were blocked in 5% milk in 1× phosphate buffered saline solution with 0.1% Tween (PBS-T). Incubation with an anti-PSD95 primary antibody (1:2000, LS-C105334, LifeSpan Biosciences) and a loading control anti-α-tubulin antibody (1:1000, MCA77G, AbD Serotec) was performed at 4 °C overnight in 1% milk in PBS-T. Secondary antibody incubation was performed for 1 h at room temperature using an anti-rabbit IRdye 800CW (LI-COR Biotechnology) and anti-rat 680 conjugated secondary antibodies (Sigma-Aldrich), both at 1:10,000 dilution, and visualised with the LI-COR Odyssey Fc.

### Imaging Statistical Analysis

The area sizes of PSDs and NCs computed by Imaris Cell were converted to average diameters assuming structures were circles. Long and short axes were determined by fitting structures to an ellipse by Imaris Cell. The aspect ratio of NCs was quantified as the ratio of the short and long axis. Average diameters were taken as the mean average and standard deviation of the mean (SD) from the median average diameters obtained from each of the 3 mice in each data set. One-way analysis of variance (ANOVA) with a *post-hoc* Tukey test were performed in SPSS (IBM SPSS Statistics, Version 21.0) to confirm specific pairwise significant differences between groups. Synapse spine diameter data were analysed by the Kruskall-Wallis test performed in GraphPad Prism (San Diego, CA, USA). Two-sample *t*-test was done to determine statistical significance of differences in the percentage of juxtaposition between PSD95 and Synaptophysin puncta.

## Additional Information

**How to cite this article**: Broadhead, M. J. *et al*. PSD95 nanoclusters are postsynaptic building blocks in hippocampus circuits. *Sci. Rep.* **6**, 24626; doi: 10.1038/srep24626 (2016).

## Supplementary Material

Supplementary Information

## References

[b1] Andersen P., Morris R., Amaral D., Bliss T., & O’Keefe J., editors. The hippocampus book. (Oxford University Press, 2007).

[b2] Harris K. M. & Landis D. M. Membrane structure at synaptic junctions in area CA1 of the rat hippocampus. Neuroscience 19, 857–872 (1986). 3796819 10.1016/0306-4522(86)90304-0

[b3] Chicurel M. E. & Harris K. M. Three-dimensional analysis of the structure and composition of CA3 branched dendritic spines and their synaptic relationships with mossy fiber boutons in the rat hippocampus. J Comp Neurol. 325, 169–182 (1992). 1460112 10.1002/cne.903250204

[b4] Schikorski T. & Stevens C. F. Quantitative ultrastructural analysis of hippocampal excitatory synapses. J Neurosci. 17, 5858–5867 (1997). 9221783 10.1523/JNEUROSCI.17-15-05858.1997 PMC6573206

[b5] Harris K. M. & Weinberg R. J. Ultrastructure of Synapses in the Mammalian Brain. Cold Spring Harb Perspect Biol. 4, doi: 10.1101/cshperspect.a012526 (2012). PMC3331701 22357909

[b6] Geinisman Y. . Structural synaptic correlate of long- term potentiation: formation of axospinous synapses with multiple, completely partitioned transmission zones. Hippocampus 3, 435 (1993). 8269035 10.1002/hipo.450030405

[b7] Geinisman Y. Structural synaptic modifications associated with hippocampal LTP and behavioral learning. Cereb. Cortex. 10, 952–962 (2000). 11007546 10.1093/cercor/10.10.952

[b8] Henze D. A., Card J. P., Barrionuevo G. & Ben-Ari Y. Large amplitude miniature excitatory postsynaptic currents in hippocampal CA3 pyramidal neurons are of mossy fiber origin. J Neurophysiol. 77, 1075 (1997). 9084583 10.1152/jn.1997.77.3.1075

[b9] Zhao S. . Structural plasticity of spines at giant mossy fiber synapses. Front Neural Circuits. 6, 103 (2012). 23264762 10.3389/fncir.2012.00103 PMC3524460

[b10] Dani A., Huang B., Bergan J., Dulac C. & Zhuang X. Superresolution Imaging of Chemical Synapses in the Brain. Neuron. 68, 843–856 (2010). 21144999 10.1016/j.neuron.2010.11.021 PMC3057101

[b11] MacGillavry H. D., Song Y., Raghavachari S. & Blanpied T. A. Nanoscale scaffolding domains within the postsynaptic density concentrate synaptic AMPA receptors. Neuron. 78, 615–622 (2013). 23719161 10.1016/j.neuron.2013.03.009 PMC3668352

[b12] Nair D. . Super-Resolution Imaging Reveals That AMPA Receptors Inside Synapses Are Dynamically Organized in Nanodomains Regulated by PSD95. J. Neurosci. 33, 13204–13224 (2013). 23926273 10.1523/JNEUROSCI.2381-12.2013 PMC6619720

[b13] Fukata Y. . Local palmitoylation cycles define activity-regulated postsynaptic subdomains. J. Cell Biol 202, 145–161 (2013). 23836932 10.1083/jcb.201302071 PMC3704990

[b14] Lu H., Macgillavry H. D., Frost N. A. & Blanpied T. A. Multiple spatial and kinetic subpopulations of CaMKII in spines and dendrites as resolved by single- molecule tracking PALM. J. Neurosci. 34, 7600–7610 (2014). 24872564 10.1523/JNEUROSCI.4364-13.2014 PMC4035521

[b15] Gray E. G. Axosomatic and axodendritic synapses in the cerebral cortex. J Anat. 93, 420–433 (1959). 13829103 PMC1244535

[b16] Cotman C. W., Banker G., Churchill L. & Taylor D. Isolation of postsynaptic densities from rat brain. J Cell Biol. 63, 441–455 (1974). 4138148 10.1083/jcb.63.2.441 PMC2110949

[b17] Carlin R. K., Grab D. J., Cohen R. S. & Siekevitz P. Isolation and characterization of postsynaptic densities from various brain regions: enrichment of different types of postsynaptic densities. J Cell Biol. 86, 831–845 (1980). 7410481 10.1083/jcb.86.3.831 PMC2110694

[b18] Bayes A. . Characterization of the proteome, diseases and evolution of the human postsynaptic density. Nat. Neurosci. 14, 19–21 (2011). 21170055 10.1038/nn.2719 PMC3040565

[b19] Chen X. . Mass of the Postsynaptic Density and Enumeration of Three Key Molecules. Proc. Natl. Acad. Sci. USA 102, 11551–11556 (2005). 16061821 10.1073/pnas.0505359102 PMC1182136

[b20] Cho K. O., Hunt C. A. & Kennedy M. B. The rat brain postsynaptic density fraction contains a homolog of the Drosophila discs-large tumor suppressor protein. Neuron. 9, 929–942 (1992). 1419001 10.1016/0896-6273(92)90245-9

[b21] Valtschanoff J. G. & Weinberg R. J. Laminar organization of the NMDA receptor complex within the postsynaptic density. J Neurosci. 21, 1211–1217 (2001). 11160391 10.1523/JNEUROSCI.21-04-01211.2001 PMC6762240

[b22] Petersen J. D. . Distribution of postsynaptic density (PSD)-95 and Ca2+/calmodulin-dependent protein kinase II at the PSD. J Neurosci. 23, 11270–11278 (2003). 14657186 10.1523/JNEUROSCI.23-35-11270.2003 PMC6741048

[b23] Chen X. . Organization of the core structure of the postsynaptic density. Proc. Natl. Acad. Sci. USA 11, 4453–4458 (2008). 18326622 10.1073/pnas.0800897105 PMC2393784

[b24] Husi H., Ward M. A., Choudhary J. S., Blackstock W. P. & Grant S. G. Proteomic analysis of NMDA receptor-adhesion protein signaling complexes. Nat. Neurosci. 3, 661–669 (2000). 10862698 10.1038/76615

[b25] Fernandez E. . Targeted tandem affinity purification of PSD-95 recovers core postsynaptic complexes and schizophrenia susceptibility proteins. Mol Syst Biol. 5, 269 (2009). 19455133 10.1038/msb.2009.27 PMC2694677

[b26] Husi H. & Grant S. G. N. Isolation of 2000-kDa complexes of N-methyl-D-aspartate receptor and postsynaptic density 95 from mouse brain. J. Neurochem. 77, 281–291 (2001). 11279284 10.1046/j.1471-4159.2001.t01-1-00248.x

[b27] Nithianantharajah J. . Synaptic scaffold evolution generated components of vertebrate cognitive complexity. Nat Neurosci. 16, 16–24 (2013). 23201973 10.1038/nn.3276 PMC4131247

[b28] Elias G. M. . Synapse-specific and developmentally regulated targeting of AMPA receptors by a family of MAGUK scaffolding proteins. Neuron. 52, 307–320 (2006). 17046693 10.1016/j.neuron.2006.09.012

[b29] Grant S. G. N. Synaptopathies: diseases of the synaptome. Curr Opin Neurobiol. 22, 522–529 (2012). 22409856 10.1016/j.conb.2012.02.002

[b30] Kirov G. . De novo CNV analysis implicates specific abnormalities of postsynaptic signalling complexes in the pathogenesis of schizophrenia. Mol Psychiatr. 17, 142–153 (2012). 10.1038/mp.2011.154 PMC3603134 22083728

[b31] Purcell S. M. . A polygenic burden of rare disruptive mutations in schizophrenia. Nature. 506, 185–190 (2014). 24463508 10.1038/nature12975 PMC4136494

[b32] Carr M. & Frank L. A single microcircuit with multiple functions: state dependent information processing in the hippocampus. Curr. Opin. Neurobiol. 22, 704–708 (2012). 22480878 10.1016/j.conb.2012.03.007 PMC3438355

[b33] Csicsvari J., Hirase H., Mamiya A. & Buzsaki G. Ensemble patterns of hippocampal CA3- CA1 neurons during sharp wave- associated population events. Neuron 28, 585–594 (2000). 11144366 10.1016/s0896-6273(00)00135-5

[b34] Chance F. Hippocampal Phase Precession from Dual Input Components. J. Neurosci. 32, 16693–16703 (2012). 23175823 10.1523/JNEUROSCI.2786-12.2012 PMC6621772

[b35] Nikonenko I. . PSD-95 Promotes Synaptogenesis and Multiinnervated Spine Formation through Nitric Oxide Signaling. J Cell Biol. 183, 1115–1127 (2008). 19075115 10.1083/jcb.200805132 PMC2600742

[b36] Kishimoto Y., Kano M., Kirino Y., Nakazawa K. & Tonegawa S. Hippocampal CA3 NMDA receptors are crucial for adaptive timing of trace eyeblink conditioned response. J Neurosci. 26, 1562–1570 (2006). 16452679 10.1523/JNEUROSCI.4142-05.2006 PMC6675508

[b37] Nägerl U. V., Köstinger G., Anderson J. C., Martin K. A. C. & Bonhoeffer T. Protracted synaptogenesis after activity- dependent spinogenesis in hippocampal neurons. J Neurosci. 27, 8149–8156 (2007). 17652605 10.1523/JNEUROSCI.0511-07.2007 PMC6672732

[b38] Friedman H. V., Bresler T., Garner C. C. & Ziv N. E. Assembly of new individual excitatory synapses: time course and temporal order of synaptic molecule recruitment. Neuron 27, 57 (2000). 10939331 10.1016/s0896-6273(00)00009-x

[b39] Okabe S., Miwa A. & Okado H. Spine formation and correlated assembly of presynaptic and postsynaptic molecules. J Neurosci. 21, 6105–6114 (2001). 11487634 10.1523/JNEUROSCI.21-16-06105.2001 PMC6763142

[b40] El-Husseini A. D. . Synaptic Strength Regulated by Palmitate Cycling on PSD-95. Cell 108, 849–863 (2002). 11955437 10.1016/s0092-8674(02)00683-9

[b41] Hruska M., Henderson N. T., Xia N. L., Marchand S. J. L. & Dalva M. B. Anchoring and synaptic stability of PSD- 95 is driven by ephrin- B3. Nat Neurosci. 18, 1594–1605 (2015). 26479588 10.1038/nn.4140 PMC5396457

[b42] Zhang P. & Lisman J. Activity- dependent regulation of synaptic strength by PSD- 95 in CA1 neurons. J Neurophysiol 107, 1058–1066 (2012). 22114157 10.1152/jn.00526.2011 PMC3289452

[b43] Elias G. M. . Synapse-specific and developmentally regulated targeting of AMPA receptors by a family of MAGUK scaffolding proteins. Neuron 52, 307–320 (2006). 17046693 10.1016/j.neuron.2006.09.012

[b44] Migaud M. . Enhanced long-term potentiation and impaired learning in mice with mutant postsynaptic density-95 protein. Nature 396, 433–439 (1998). 9853749 10.1038/24790

[b45] Carlisle H. J., Fink A. E., Grant S. G. N. & O’Dell T. J. Opposing effects of PSD-93 and PSD-95 on long-term potentiation and spike timing-dependent plasticity. J Physiology 586, 5885–5900 (2008). 10.1113/jphysiol.2008.163469 PMC2655416 18936077

[b46] Spruston N. Distant synapses raise their voices. Nat Neurosci. 3, 849–851 (2000). 10966607 10.1038/78734

[b47] Nicholson D. A. . Distance- Dependent Differences in Synapse Number and AMPA Receptor Expression in Hippocampal CA1 Pyramidal Neurons. Neuron 50, 431–442 (2006). 16675397 10.1016/j.neuron.2006.03.022

[b48] Stewart M. G. . Chemically induced long- term potentiation increases the number of perforated and complex postsynaptic densities but does not alter dendritic spine volume in CA1 of adult mouse hippocampal slices. Eur. J. Neurosci. 21, 3368–3378 (2005). 16026474 10.1111/j.1460-9568.2005.04174.x

[b49] Nieto-Sampedro M., Hoff S. F. & Cotman C. W. Perforated Postsynaptic Densities: Probable Intermediates in Synapse Turnover. Proc. Natl. Acad. Sci. USA 79(18), 5718–5722 (1982). 6957887 10.1073/pnas.79.18.5718 PMC346976

[b50] Geinisman Y., de Toledo-Morrell L. & Morrell F. Loss of perforated synapses in the dentate gyrus: morphological substrate of memory deficit in aged rats. Proc. Natl. Acad. Sci. USA 83, 3027–3031 (1986). 3458260 10.1073/pnas.83.9.3027 PMC323440

[b51] Dosemeci A. . Composition of the synaptic PSD-95 complex. Mol Cell Proteomics. 6, 1749–1760 (2007). 17623647 10.1074/mcp.M700040-MCP200 PMC2096750

[b52] Emes R. D. . Evolutionary expansion and anatomical specialization of synapse proteome complexity. Nat Neurosci 11, 799–806 (2008). 18536710 10.1038/nn.2135 PMC3624047

[b53] Geinisman Y. Perforated axospinous synapses with multiple, completely partitioned transmission zones - probable structural intermediates in synaptic plasticity. Hippocampus 3, 417–434 (1993). 8269034 10.1002/hipo.450030404

[b54] Toni N. . Remodeling of synaptic membranes after induction of long- term potentiation. J Neurosci. 21, 6245–6251 (2001). 11487647 10.1523/JNEUROSCI.21-16-06245.2001 PMC6763190

[b55] McKinney S. A., Murphy C. S., Hazelwood K. L., Davidson M. W. & Looger L. L. A bright and photostable photoconvertible fluorescent protein. Nat. Methods. 6, 131–133 (2009). 19169260 10.1038/nmeth.1296 PMC2745648

[b56] Zhou D., Ren J.-X., Ryan T. M., Higgins N. P. & Townes T. M. Rapid tagging of endogenous mouse genes by recombineering and ES cell complementation of tetraploid blastocysts. Nucleic Acids Res. 32, e128 (2004). 15356288 10.1093/nar/gnh128 PMC519128

[b57] Kopanitsa M. V., Afinowi N. O. & Grant S. G. N. Recording long-term potentiation of synaptic transmission by three-dimensional multi-electrode arrays. BMC Neurosci. 7, 61 (2006). 16942609 10.1186/1471-2202-7-61 PMC1574331

[b58] Andersen P., Sundberg S. H., Sveen O. & Wigstrom H. Specific long-lasting potentiation of synaptic transmission in hippocampal slices. Nature. 266, 736–737 (1977). 195210 10.1038/266736a0

[b59] Benavides-Piccione R., Fernaud-Espinosa I., Robles V., Yuste R. & DeFelipe J. Age-Based Comparison of Human Dendritic Spine Structure Using Complete Three-Dimensional Reconstructions. Cereb Cortex. 23, 1798–1810 (2013). 22710613 10.1093/cercor/bhs154 PMC3698364

[b60] Elston G. N. & Rosa M. G. The occipitoparietal pathway of the macaque monkey: comparison of pyramidal cell morphology in layer III of functionally related cortical visual areas. Cereb Cortex. 7, 432–452 (1997). 9261573 10.1093/cercor/7.5.432

[b61] O’Neil A., Prevelige P. E., Basu G. & Douglas T. Coconfinement of Fluorescent Proteins: Spatially Enforced Communication of GFP and mCherry Encapsulated within the P22 Capsid. Biomacromolecules 13, 3902–3907 (2012). 23121071 10.1021/bm301347x

[b62] Gould T., Verkhusha V. & Hess S. Imaging Biological Structures with Fluorescence Photoactivation Localization Microscopy. Nat. Protoc. 4, 291–308 (2009). 19214181 10.1038/nprot.2008.246 PMC2908010

[b63] Sander J., Ester M., Kriegel H.-P. & Xu X. Density-Based Clustering in Spatial Databases: The Algorithm GDBSCAN and Its Applications. Data Min Knowl Discov. 2, 169–194 (1998).

[b64] Baddeley A. & Turner R. spatstat: An R package for analyzing spatial point patterns. J Stat Softw. 12, 1–42 (2005).

[b65] Illian J. Statistical analysis and modelling of spatial point pattern. Ch. 7, 455–457 (John Wiley, 2008).

[b66] Huttner W. B., Schiebler W., Greengard P. & De Camilli P. Synapsin I (Protein I), a Nerve Terminal-Specific Phosphoprotein. III. Its Association with Synaptic Vesicles Studied in a Highly Purified Synaptic Vesicle Preparation. J Cell Biol. 96, 1374–1388 (1983). 6404912 10.1083/jcb.96.5.1374 PMC2112660

[b67] Grant S. G., Marshall M. C., Page K. L., Cumiskey M. A. & Armstrong J. D. Synapse proteomics of multiprotein complexes: en route from genes to nervous system diseases. Hum Mol Genet. 14, 2005 Oct 15; Spec No. 2:R225-34. Epub 2005 Sep 8. 10.1093/hmg/ddi330 16150739

[b68] Frank R. A., McRae A. F., Pocklington A. J., van de Lagemaat L. N., Navarro P., Croning M. D., Komiyama N. H., Bradley S. J., Challiss R. A., Armstrong J. D., Finn R. D., Malloy M. P., MacLean A. W., Harris S. E., Starr J. M., Bhaskar S. S., Howard E. K., Hunt S. E., Coffey A. J., Ranganath V., Deloukas P., Rogers J., Muir W. J., Deary I. J., Blackwood D. H., Visscher P. M. & Grant S. G. Clustered coding variants in the glutamate receptor complexes of individuals with schizophrenia and bipolar disorder. PLoS One. 6(4), e19011 20 April 2011; doi: 10.1371/journal.pone.0019011. 21559497 PMC3084736

