# Supplementary material for: PSD95 nanoclusters are postsynaptic building blocks in hippocampus circuits
Source: Sci Rep. 2016 Apr 25;6:24626. doi: 10.1038/srep24626 (PMC4842999; doi:10.1038/srep24626)
Supplement: Supplementary Information [file srep24626-s1.pdf]

PSD95 nanoclusters are postsynaptic building blocks in hippocampus circuits.

Supplementary Information

Matthew J. Broadhead[1,2], Mathew H. Horrocks[3], Fei Zhu[1], Leila Muresan[4], Ruth Benavides-Piccione[5], Javier DeFelipe[5], David Fricker[6], Maksym V. Kopanitsa[6], Rory R. Duncan[2,7], David Klenerman[3], Noboru H. Komiyama[1], Steven F. Lee[3*], Seth G.N. Grant[1*].

1. Centre for Clinical Brain Sciences (CCBS), University of Edinburgh, Edinburgh, United Kingdom,
2. Edinburgh Super-Resolution Imaging Consortium (ESRIC), Heriot Watt University, Edinburgh, United Kingdom.
3. Department of Chemistry, University of Cambridge, Lensfield Road, Cambridge, CB2 1EW. United Kingdom.
4. Cambridge Advanced Imaging Centre (CAIC), University of Cambridge, Cambridge, United Kingdom.
5. Instituto Cajal (CSIC), Madrid, Spain.
6. Synome Ltd, Babraham Research Campus, Cambridge, United Kingdom.
7. Institute of Biological Chemistry, Biophysics and Bioengineering, Heriot-Watt University, Edinburgh, EH14 4AS, UK.

* These authors co-supervised the project.

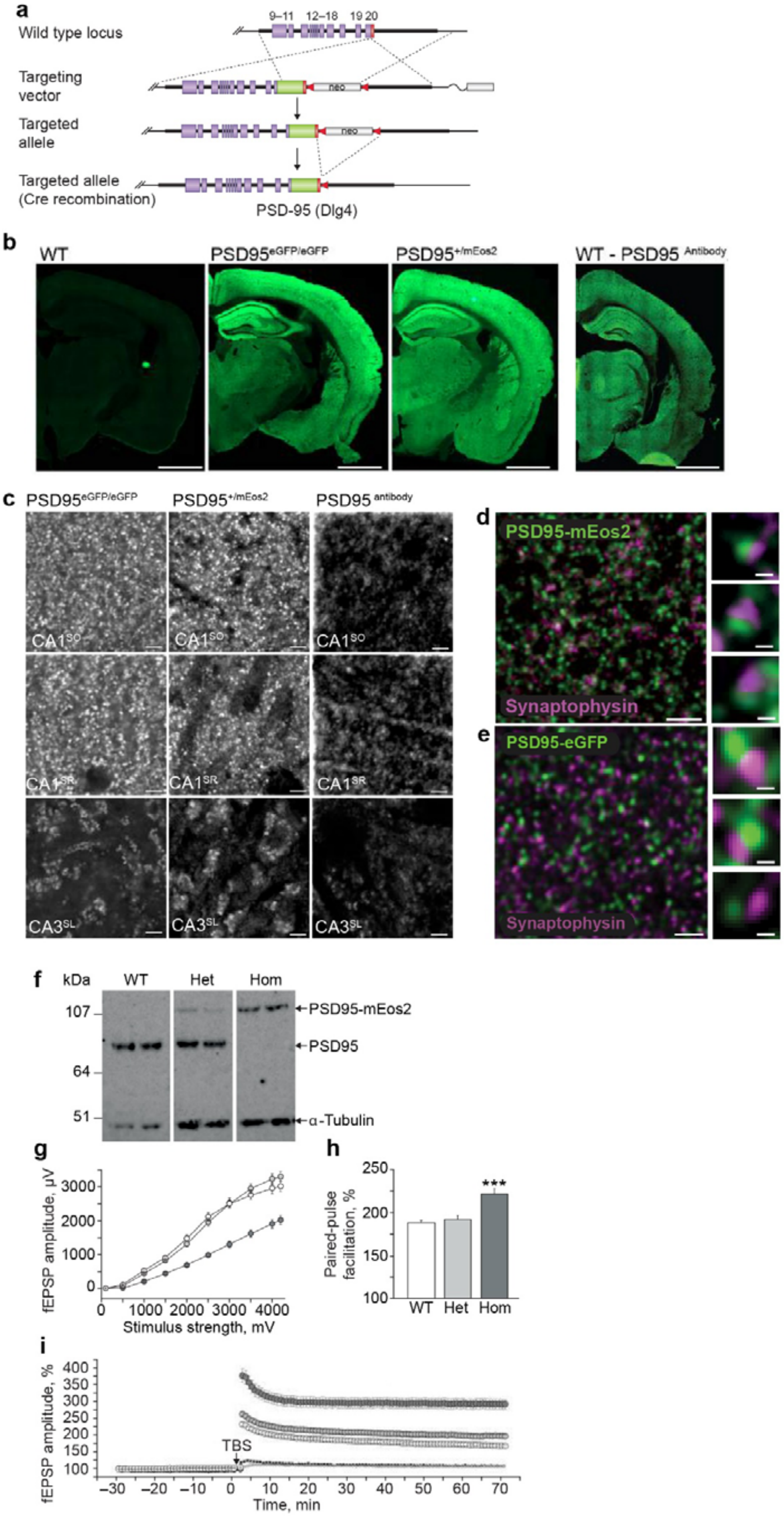
a
Wild type locus
9–11
12–18
19
20
Targeting vector
neo
Targeted allele
neo
Targeted allele (Cre recombination)
PSD-95 (Dlg4)
b
WT
PSD95$^{eGFP/eGFP}$
PSD95$^{+/mEos2}$
WT - PSD95 ^{Antibody}
c
PSD95$^{eGFP/eGFP}$
PSD95$^{+/mEos2}$
PSD95 ^{antibody}
CA1^{SO}
CA1^{SR}
CA3^{SL}
d
PSD95-mEos2
Synaptophysin
e
PSD95-eGFP
Synaptophysin
f
kDa
WT
Het
Hom
107
64
51
PSD95-mEos2
PSD95
α-Tubulin
g
fEPSP amplitude, μV
Stimulus strength, mV
h
Paired-pulse facilitation, %
WT
Het
Hom
***
i
fEPSP amplitude, %
TBS
Time, min

Supplementary Figure 1. Targeting strategy for construction of PSD95-mEos2 knockin mouse.

a. Schematic diagram of the gene targeting strategy. Wild type gene structure is shown including terminal exon (red), targeting vector and targeted allele. The neomycin-resistance cassette was excised by Cre recombination. Dark boxes, exons; green box, mEos2; red triangles, loxP sites; DTA, diphtheria toxin cassette; neo, neomycin-resistance cassette.
b. Low magnification (20×) scanning of hemi-coronal brain sections from (left to right) a WT brain section (negative) and homozygous PSD95-eGFP mouse, heterozygous PSD95-mEos2 mouse and a WT section immunostained with an anti-PSD95 antibody. WT and PSD95-eGFP sections captured at the same illumination intensity and exposure time for comparison; PSD95-mEos2 and $PSD95^{antibody}$ contrast enhanced for expression pattern comparison. Scale bar 2 mm.
c. High magnification (100×) confocal imaging of PSD95-eGFP, PSD95-mEos2 and $PSD95^{antibody}$ in three hippocampal subregions Scale bar 2 μm.
d. High magnification in the $CA1^{SR}$ of PSD95-mEos2 co-stained for synaptophysin revealing pre and postsynaptic puncta, with example inset images of synapses. Scale bar 1 μm, inset scale bars 200 nm.
e. High magnification in the $CA1^{SR}$ of PSD95-eGFP co-stained for synaptophysin revealing pre and postsynaptic puncta, with example inset images of synapses. Scale bar 1 μm, inset scale bars 200 nm.
f. Immunoblot for PSD95 with tubulin loading control from crude synaptosome extracts of WT, heterozygous and homozygous PSD95-mEos2 mice. Schematic diagrams of the *Dlg4* gene encoding the PSD95 protein. mEos2 is targeted to the C-terminal locus of PSD95. Neomycin cassette is removed from the genome following cre-recombination.
g. Baseline synaptic transmission was significantly affected by genotype ($F(2, 22.078) = 16.21$; $P = 0.00005$). Input-output relationships illustrate averaged peak fEPSP amplitudes in slices from $PSD\text{-}95^{mEos2/mEos2}$ (n = 24 slices; N = 7 mice), $PSD95^{+/mEos2}$ (n = 34; N = 11) and WT mice (n = 34; N = 9) in response to stimulation of Schäffer collaterals by biphasic voltage pulses of 0.1 – 4.2 V. Post hoc Dunnett's Multiple Comparison test demonstrated decreased AUCI/O values in PSD-95mEos2/mEos2 mice ($q = 4.496$; $P < 0.001$) compared to AUCI/O values in WT mice but no difference was found between $PSD\text{-}95^{+/mEos2}$ and WT mice ($q = 0.095$; $P < 0.05$).
WT, white symbols; heterozygous, grey symbols; homozygous, dark symbols.
h. Short term plasticity (PPF) was significantly affected by genotype ($F(2, 21.43) = 13.43$; $P = 0.00017$). *Post hoc* Dunnett's Multiple Comparison test demonstrated increased PPF values in $PSD\text{-}95^{mEos2/mEos2}$ mice ($q = 4.233$; $P < 0.001$) compared to AUCI/O values in WT mice, while no statistical difference was found between heterozygous $PSD95^{+/mEos2}$ and WT mice ($q = 0.1378$; $P > 0.05$).
i. Theta-burst stimulation (TBS) induced LTP was significantly affected by genotype ($F(2, 20.869) = 43.376$; $P < 0.0001$). *Post hoc* Dunnett's Multiple Comparison test demonstrated that LTP was significantly higher in both $PSD95^{mEos2/mEos2}$ ($q = 9.888$; $P < 0.001$) and $PSD\text{-}95^{+/mEos2}$ mutants ($q = 2.382$; $P < 0.05$) compared to WTs. Graphs in panels a–c display data expressed as the mean ± standard error of the mean.
WT, white symbols; heterozygous, grey symbols; homozygous, dark symbols.

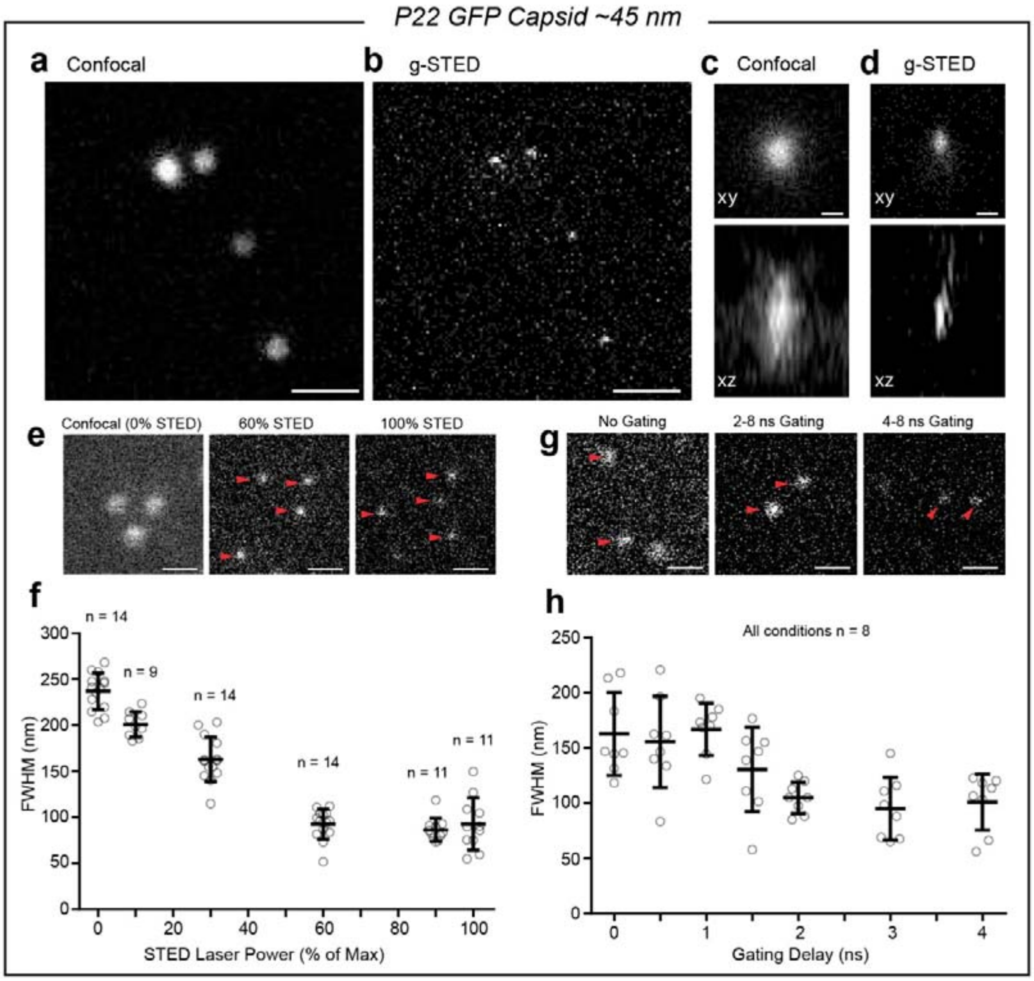

Supplementary Figure 2. Calibrating g-STED microscopy parameters using nanobeads

a. P22 capsids imaged with confocal microscopy. Scale bar 500 nm
b. P22 capsids imaged with g-STED microscopy at 80% of STED power and 2–8 ns gating. Scale bar 500 nm.
c. 3D z-stack image of P22 capsid acquired confocal microscopy. Scale bar 200 nm.
d. 3D z-stack image of P22 capsid acquired with g-STED microscopy using 40% STED power and 2–8 ns gating. Scale bar 200 nm.
e. The effect of STED laser power was tested on different capsids to determine the resolution capacity. Scale bar 500 nm.
f. FWHMs of capsids were measured to assess resolution by the size of capsids imaged with different depletion laser powers. Note that 0% STED laser power condition was a confocal image without gating, while other images included gating 2–8 ns. n = number of capsids analysed.

g. Gating delay was tested on different capsids to assess the impact of resolution. Scale bar 500 nm

h. FWHMs were measured to assess the impact on resolution.

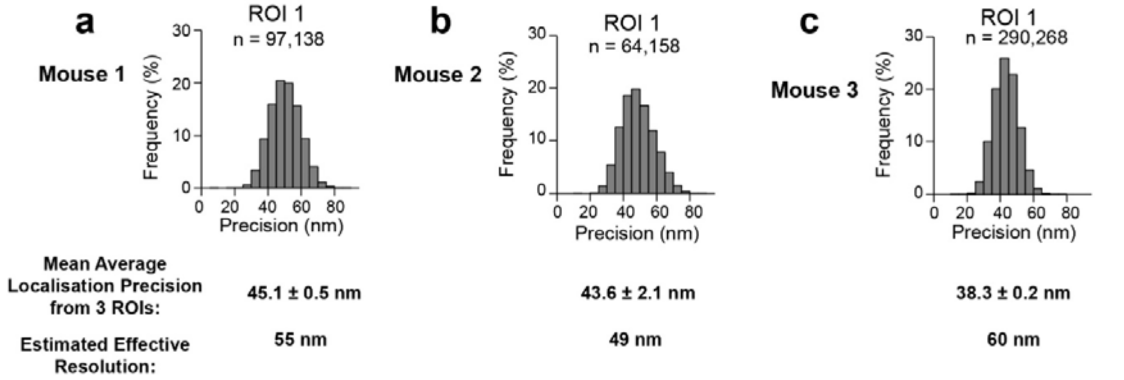

Supplementary Figure 3. Assessing effective resolution of PALM on PSD95-mEos2 brain sections.

a-c. Localisation precision error plotted in histograms from images from $CA1^{SO}$ in 3 different mice. N = number of localisation events within a whole image sequence. Small differences were noted in the mean average localisation precision averaged across 3 images from within each mice (mean ± standard deviation). Taking into account the precision fitting and the mean average NN value for each detected NC, the effective lateral resolution for PSD95-mEos2 imaging can vary between brain samples from 50 to 60 nm.

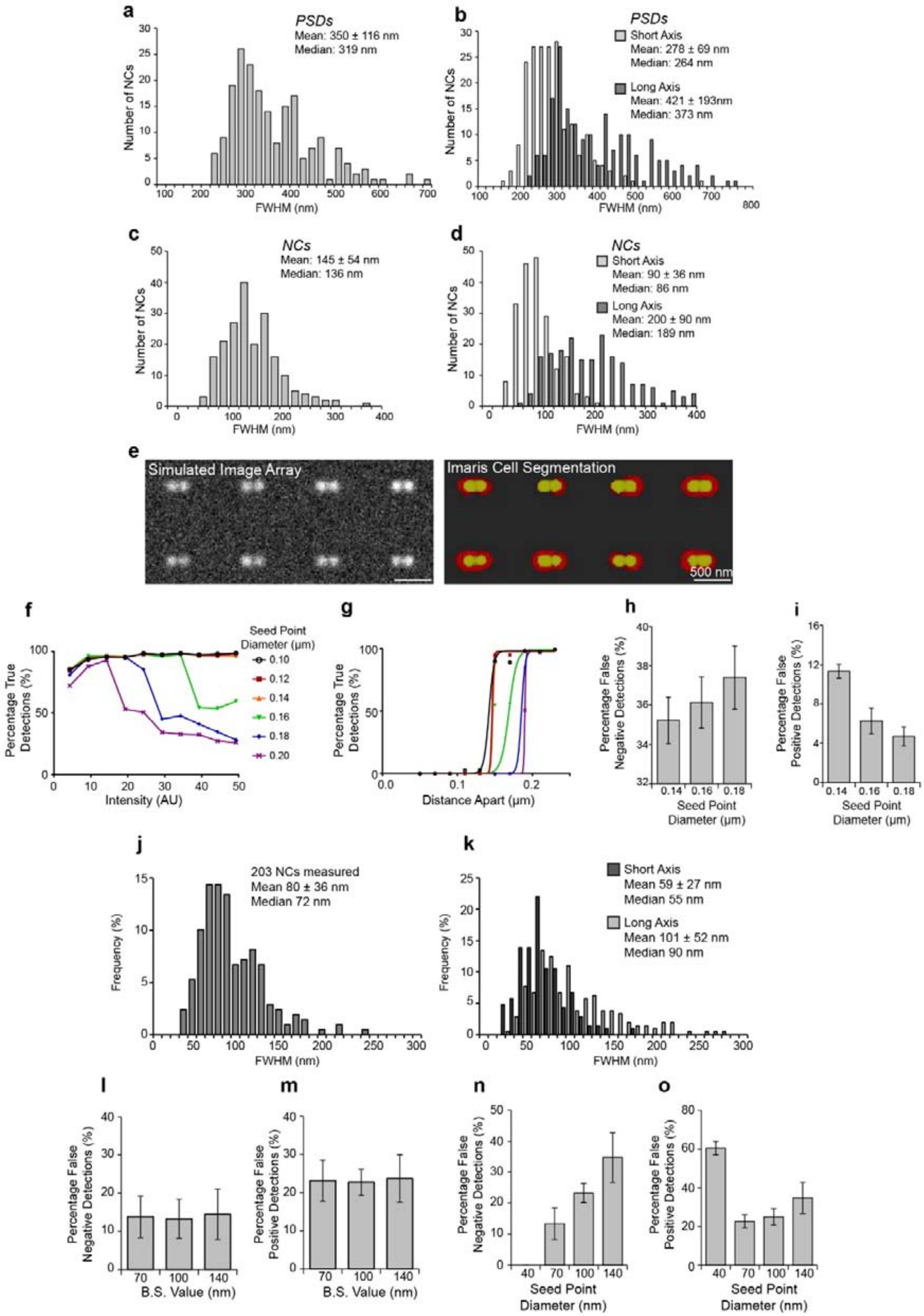
a
PSDs
Mean: 350 ± 116 nm
Median: 319 nm
Number of NCs
FWHM (nm)
b
PSDs
Short Axis
Mean: 278 ± 69 nm
Median: 264 nm
Long Axis
Mean: 421 ± 193nm
Median: 373 nm
c
NCs
Mean: 145 ± 54 nm
Median: 136 nm
d
NCs
Short Axis
Mean: 90 ± 36 nm
Median: 86 nm
Long Axis
Mean: 200 ± 90 nm
Median: 189 nm
e
Simulated Image Array
Imaris Cell Segmentation
500 nm
f
Seed Point Diameter (µm)
0.10
0.12
0.14
0.16
0.18
0.20
Percentage True Detections (%)
Intensity (AU)
g
Distance Apart (µm)
h
Percentage False Negative Detections (%)
Seed Point Diameter (µm)
i
Percentage False Positive Detections (%)
j
203 NCs measured
Mean 80 ± 36 nm
Median 72 nm
Frequency (%)
k
Short Axis
Mean 59 ± 27 nm
Median 55 nm
Long Axis
Mean 101 ± 52 nm
Median 90 nm
l
B.S. Value (nm)
m
n
Seed Point Diameter (nm)
o

Supplementary Figure 4. FWHM analysis of PSD95 structures, and validation of Imaris Cell analysis parameters.

a. Frequency histogram of the diameter of 200 PSD95-eGFP PSDs from the CA1^{SO} determined from FWHM analysis.

b. Frequency histogram of PSD short and long axes from PSD95-eGFP PSDs.

c. Frequency histogram of NC diameter from PSD95-eGFP PSDs.

d. Frequency histogram of NC short and long axes from PSD95-eGFP PSDs.

e. Example of simulated array of 2NC-PSDs with subsequent Imaris Cell segmentation of the NC-like substructures (yellow) within the overall realms of the PSDs (red).

f. Intensity of NCs was titrated to test whether different SNRs would affect image analysis. Graph plots true detections of simulated NCs (%) using different seed point diameter values with different intensity / SNR values.

g. The distance between NCs within each pair was titrated from 0.05 µm to 0.23 µm. **f**. Graph plots true positive detections (%) using different seed point diameter values with different separation distances. Points are fitted to sigmoidal curves.

h. Graph plots false negative detections (%) using Imaris Cell assessed against manually detected PSD95-eGFP NCs using different seed point diameters.
i. Graph plots false positive detections (%) using Imaris Cell assessed against manually detected PSD95-eGFP NCs using different seed point dimeters.
j. Frequency histogram of the diameter of 200 PSD95-mEos2 NCs from the CA1^{SO} determined from FWHM analysis.
k. Frequency histogram of the long and short axes of NCs from FWHM analysis.
l. Different background subtraction (B.S.) diameters were tested and the percentages of false negative detections with Imaris Cell were assessed against true positive detections from manual quantification.
m. The percentage of false positive detections with Imaris Cell against true positive detections with manual quantifications were assessed for different B.S. diameters.
n. Graph plots false negative detections (%) using Imaris Cell assessed against manually detected PSD95-mEos2 NCs using different seed point diameters.
o. Graph plots false positive detections (%) using Imaris Cell assessed against manually detected PSD95-mEos2 NCs using different seed point dimeters.

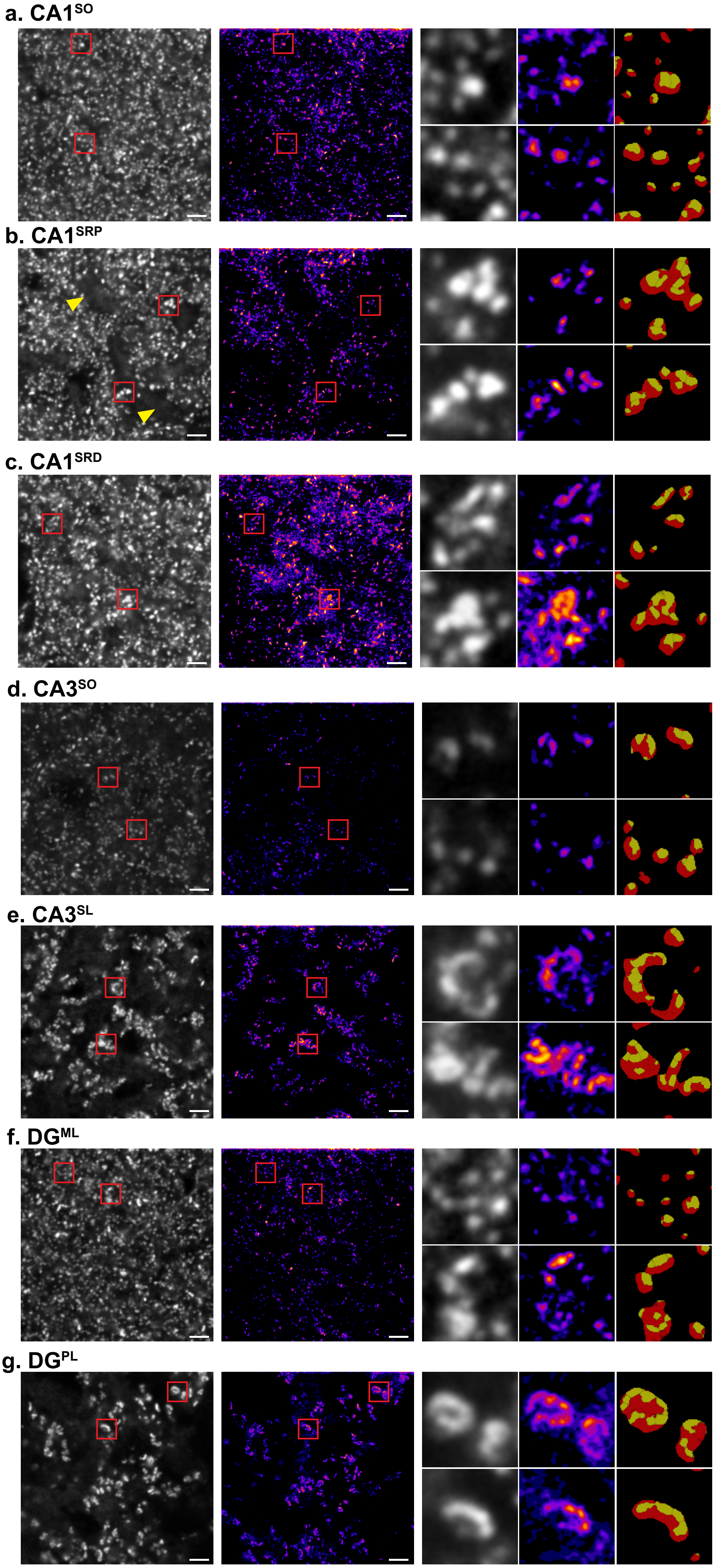
a. CA1SO
b. CA1SRP
c. CA1SRD
d. CA3SO
e. CA3SL
f. DGML
g. DGPL

Supplementary Figure 5. Catalogue of g-STED images from hippocampal sub-regions.

Confocal and g-STED images taken from each of the seven sub-regions (a-g), each with two pairs of inset images (2 μm x 2 μm), are displayed along with Imaris Cell segmentations. Scale Bars 2 μm. Note yellow arrows in the $CA1^{SRP}$ highlight possible fibre tracts from nearby pyramidal neurons.

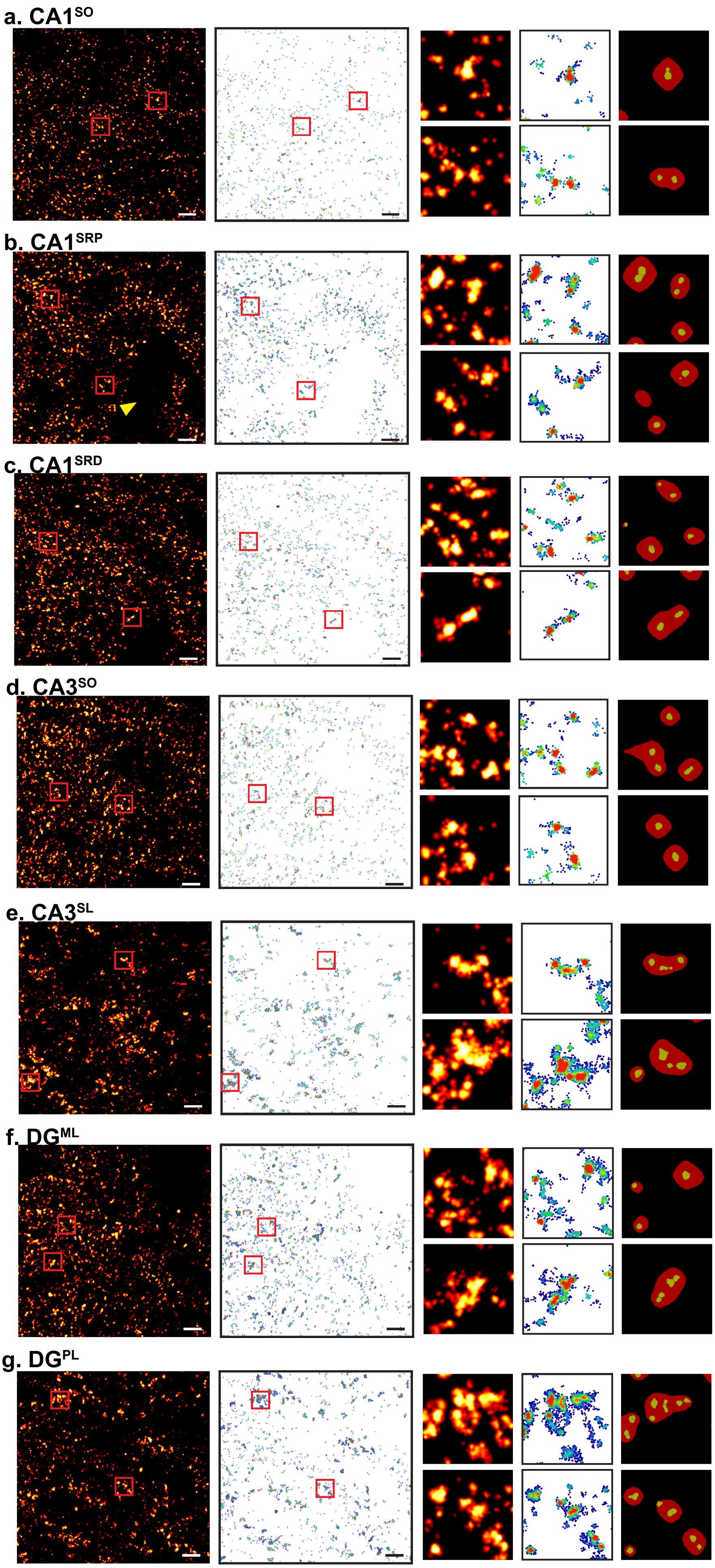
a. CA1SO
b. CA1SRP
c. CA1SRD
d. CA3SO
e. CA3SL
f. DGML
g. DGPL

Supplementary Figure 6. Catalogue of PALM images from hippocampal sub-regions.

PALM and NN images taken from each of the seven sub-regions (a-g), each with two pairs of inset images (2 µm x 2 µm), are displayed along with Imaris Cell segmentations. Scale Bars 2 µm. Note yellow arrows in the $CA1^{SRP}$ highlight possible fibre tracts from nearby pyramidal neurons.

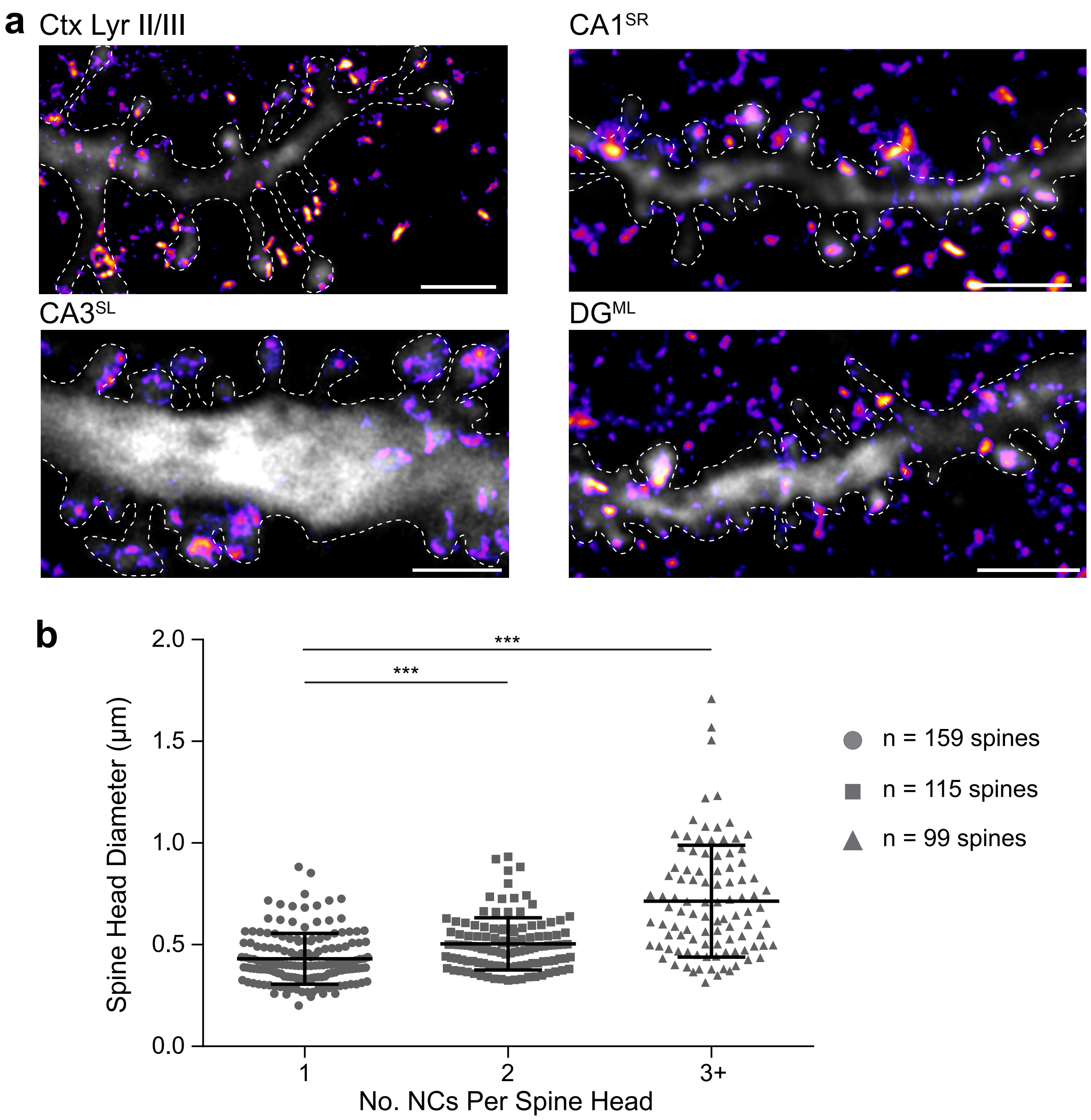

Supplementary Figure 7. G-STED of PSD95-eGFP in dye-filled neurons.

a. Example of a dendrite from a dye filled pyramidal neuron from cortical layer II/III, CA1 pyramidal neuron dendrite in the $CA1^{SR}$, CA3 pyramidal neuron dendrite in the $CA3^{SL}$ and a granule cell dendrite in the DG^{ML}. Scale bars 2 µm.

b. Spine head diameter positively correlated with the number of PSD95-eGFP NCs expressed within the spine. Quantifications pooled from across all spines of neurons. Kruskall-Wallis test with pairwise comparison test confirmed statistically significant differences in spine diameters.

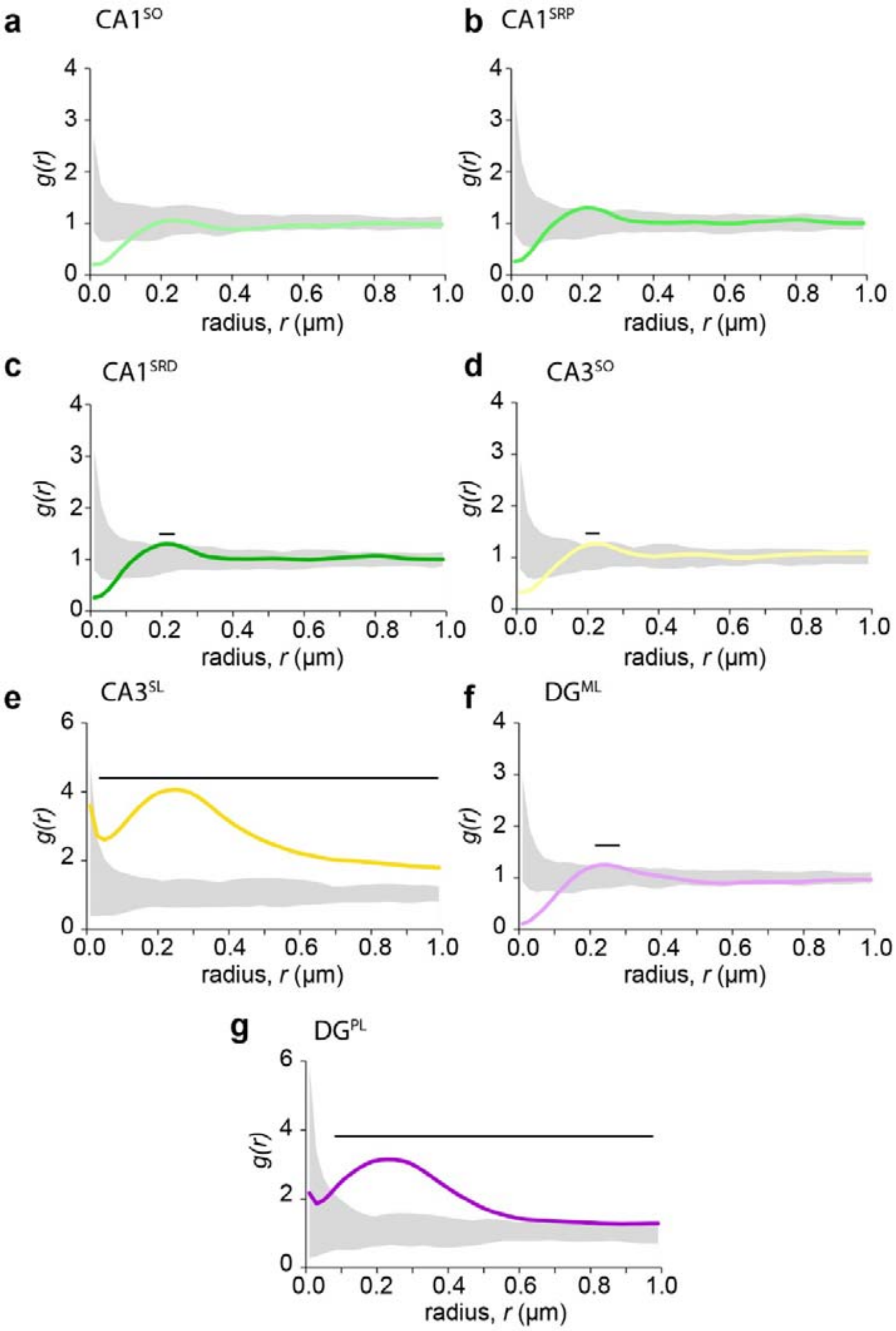

Supplementary Figure 8. Spatial analysis of NCs using paired correlation function analysis

Paired correlation function (g(r)) analysis on the spatial distribution of PSD95-eGFP NCs, plotted (g(r)) over a radius of 1 μm. Each of the seven sub-regions (a–g) are plotted (colour coded as per Fig.2a). Grey fields represent upper and lower confidence limits of spatial randomness for each respective sub-region. Black lines indicate where NCs show statistically significant clustering over their respective envelopes.

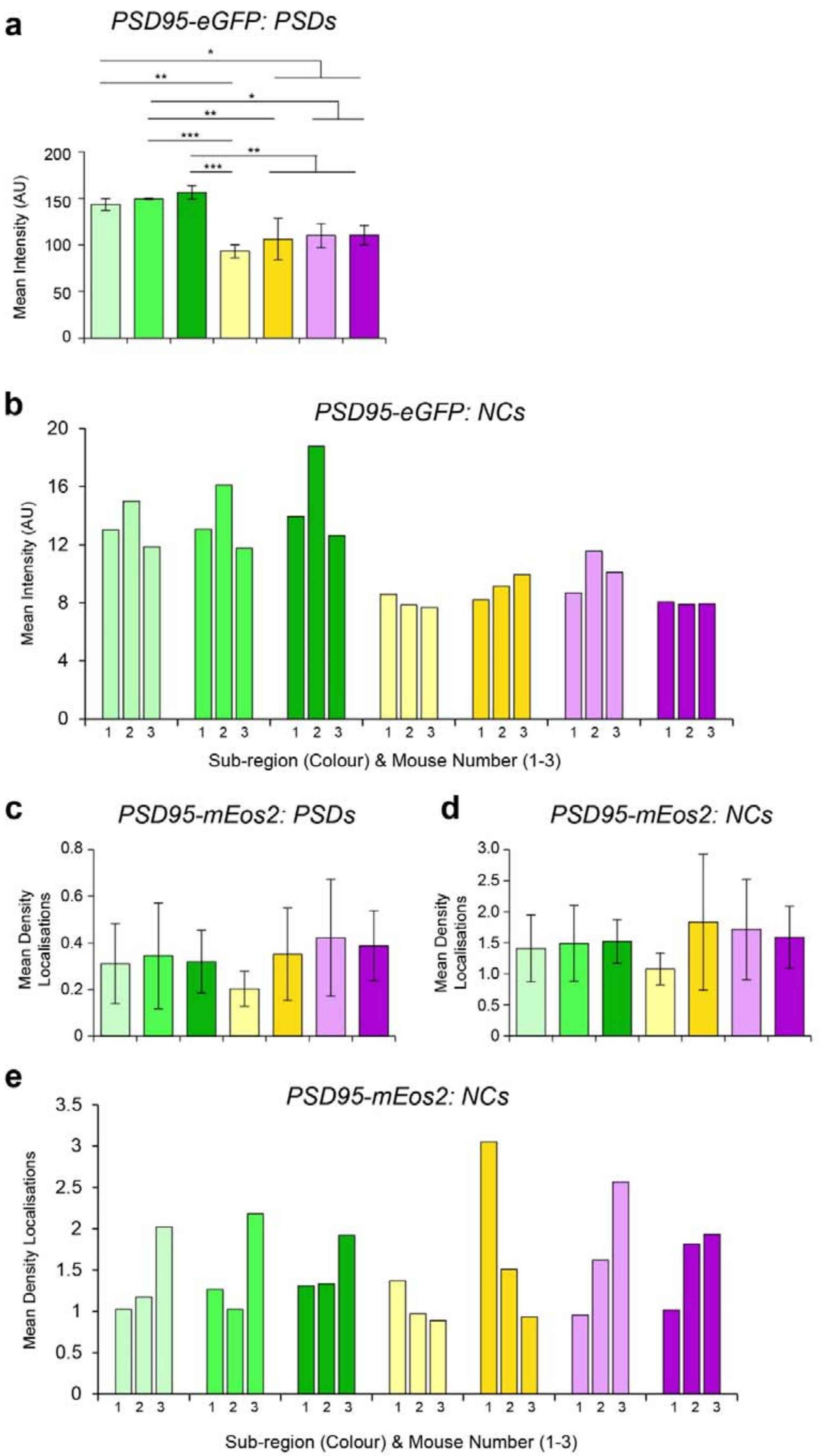
a
PSD95-eGFP: PSDs
*
**
*
**
***
**
***
Mean Intensity (AU)
200
150
100
50
0
b
PSD95-eGFP: NCs
Mean Intensity (AU)
20
16
12
8
4
0
1 2 3
Sub-region (Colour) & Mouse Number (1-3)
c
PSD95-mEos2: PSDs
Mean Density Localisations
0.8
0.6
0.4
0.2
0
d
PSD95-mEos2: NCs
Mean Density Localisations
3.0
2.5
2.0
1.5
1.0
0.5
0
e
PSD95-mEos2: NCs
Mean Density Localisations
3.5
3
2.5
2
1.5
1
0.5
0
1 2 3
Sub-region (Colour) & Mouse Number (1-3)

Supplementary Figure 9. Mean fluorescence intensities and localisation densities.

a. Bar chart of the mean fluorescence intensity of PSD95-eGFP PSDs from each sub-region (colour coded as per Fig.2a)

b. Bar chart of the mean fluorescence intensity of PSD95-eGFP NCs from each sub-region (colour coded) and each mouse brain section (1–3). Relative differences between sub-regions were observed in each of the three mouse brain sections.

c. Bar chart of the mean density of PSD localisations from each sub-region in PSD95-mEos2 brain sections.

d. Bar chart of the mean density of NC localisations from each sub-region in PSD95-mEos2 brain sections.

e. Bar chart of the mean density of PSD localisations from each sub-region in PSD95-mEos2 mouse brain section (1–3). Variability between the samples masks any significant trends of sub-regional differences in localisations per NC.

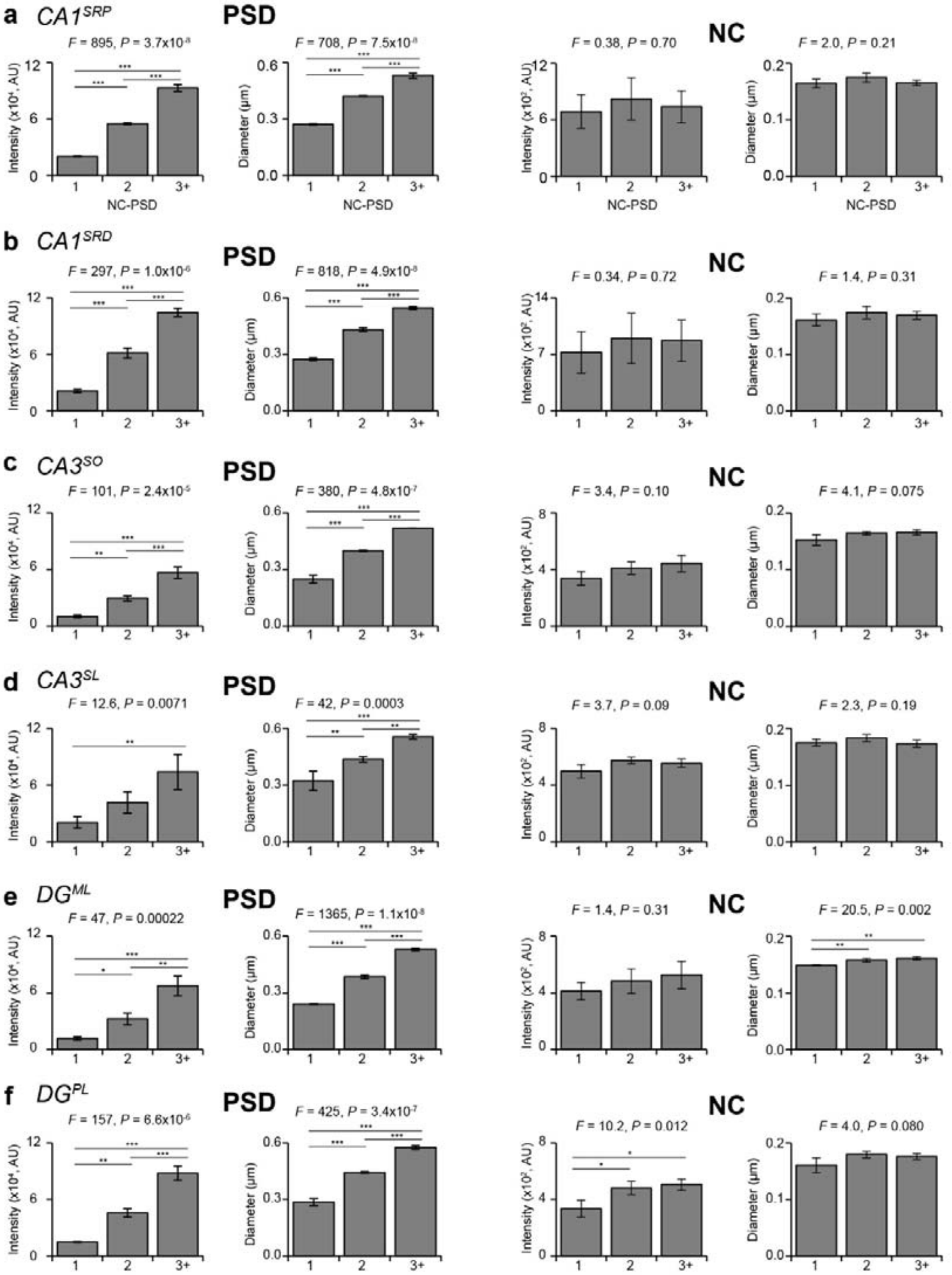

Supplementary Figure 10. The conserved organisational principle of PSD-95 across the hippocampus as revealed by g-STED.

The average integrated fluorescence intensity and average diameter of PSDs and NCs from the three synapse subtypes (1, 2, 3+NC-PSDs) from six sub-regions (**a–f**; excluding the $CA1^{SO}$). Every sub-region appears to show that there is a positive correlation between PSD size/fluorescence intensity and the number of NCs, while the size and intensity of the NCs is

independent of the synapse subtype. Only two exceptions to this were noted: in the DG^{ML} NCs composed of 2 and 3+NC-PSDs were larger than those composed of 1NC-PSDs (P < 0.01) and in the DG^{PL} NCs composed of 2 and 3+NC-PSDs were greater in fluorescence intensity than those of 1NC-PSDs (P < 0.05).

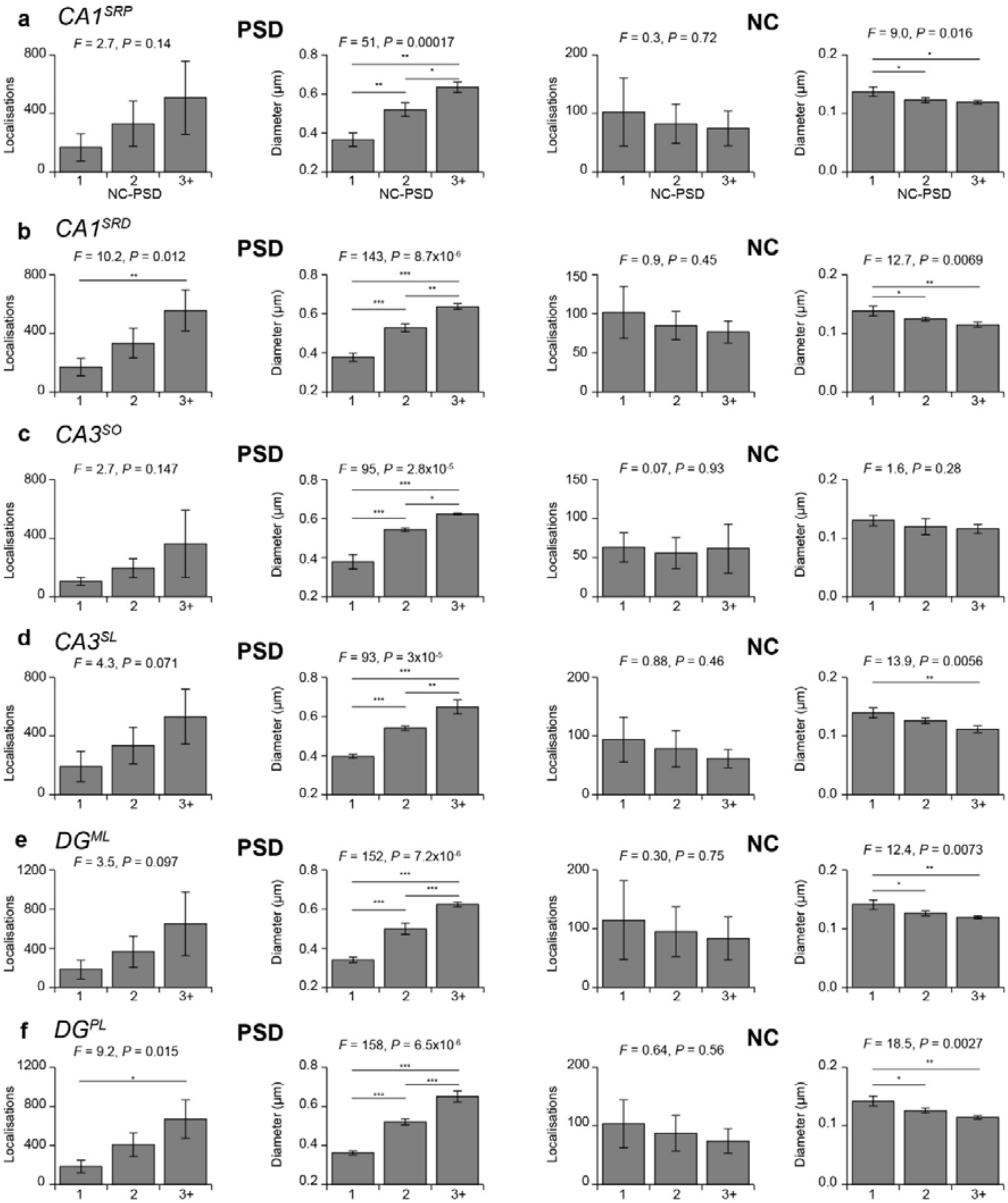

Supplementary Figure 11. The conserved organisational principle of PSD-95 across the hippocampus as revealed by g-STED.

The average total numbers of localisations and average diameters of PSDs and NCs from the three synapse subtypes (1, 2, 3+NC-PSDs) from six sub-regions (a–f). While there is a trend for PSDs to contain a greater number of localisation events with a larger number of NCs, this was not statistically significant due to a considerable variability in the total number of localisations from section to section. Although NCs became statistically significantly

smaller in every sub-region except the CA3^{SO}, the extent of significance was not as great as the differences observed in PSDs sizes.
